# Molecular Engineering of Metalloporphyrins for High‐Performance Energy Storage: Central Metal Matters

**DOI:** 10.1002/cssc.202202090

**Published:** 2023-01-13

**Authors:** Shirin Shakouri, Ebrahim Abouzari‐Lotf, Jie Chen, Thomas Diemant, Svetlana Klyatskaya, Frank Dieter Pammer, Asato Mizuno, Maximilian Fichtner, Mario Ruben

**Affiliations:** ^1^ Institute of Nanotechnology Karlsruhe Institute of Technology P.O. Box 3640 76021 Karlsruhe Germany; ^2^ Helmholtz Institute Ulm (HIU) Electrochemical Energy Storage Helmholtzstraße 11 Ulm 89081 Germany; ^3^ Institute for Quantum Materials and Technologies (IQMT) Karlsruhe Institute of Technology P.O. Box 3640 76021 Karlsruhe Germany; ^4^ Centre Européen de Science Quantique (CESQ) Institut de Science et d'Ingénierie Supramoléculaires (ISIS) Université de Strasbourg 8, Allée Gaspard Monge 67000 Strasbourg France

**Keywords:** organic electrodes, metalloporphyrin, multi-electron redox reactions, porphyrin metal centers, structure–performance relationships

## Abstract

Porphyrin derivatives represent an emerging class of redox‐active materials for sustainable electrochemical energy storage. However, their structure–performance relationship is poorly understood, which confines their rational design and thus limits access to their full potential. To gain such understanding, we here focus on the role of the metal ion within porphyrin molecules. The A_2_B_2_‐type porphyrin 5,15‐bis(ethynyl)‐10,20‐diphenylporphyrin and its first‐row transition metal complexes from Co to Zn are used as models to investigate the relationships between structure and electrochemical performance. It turned out that the choice of central metal atom has a profound influence on the practical voltage window and discharge capacity. The results of DFT calculations suggest that the choice of central metal atom triggers the degree of planarity of the porphyrin. Single crystal diffraction studies illustrate the consequences on the intramolecular rearrangement and packing of metalloporphyrins. Besides the direct effect of the metal choice on the undesired solubility, efficient packing and crystallinity are found to dictate the rate capability and the ion diffusion along with the porosity. Such findings open up a vast space of compositions and morphologies to accelerate the practical application of resource‐friendly cathode materials to satisfy the rapidly increasing need for efficient electrical energy storage.

## Graphical abstract

**Centered around the center**: An A_2_B_2_‐type porphyrin is employed as a base cathode structure to visualize the effect of centrally‐coordinated divalent metal ion of first‐row transition metals from Co to Zn on their charge‐storage performance. A comparative study shows that the choice of the central metal atom directly influences the undesired solubility, crystallinity, porosity, and conductivity and dictates the energy‐storage performance.

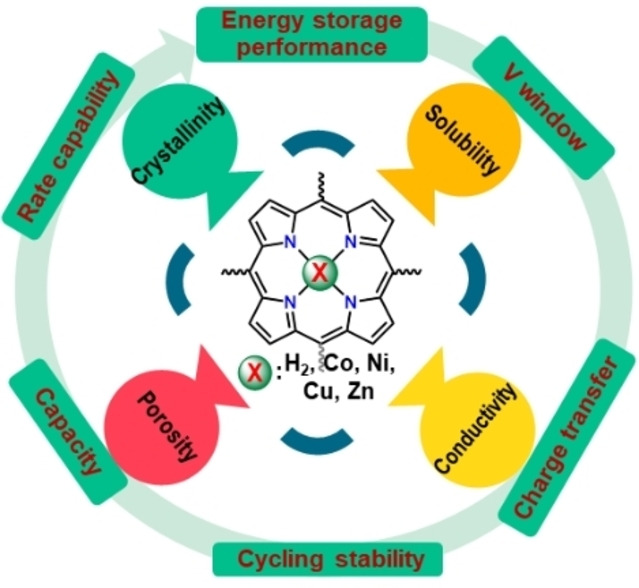

## Introduction

Electrochemical energy storage based on organic electrodes offers promising opportunities to further improve the existing technologies, create innovative systems, and pose valid options in terms of environmental footprint.[^1^, ^2^, ^3^] Several general classes of redox‐active organics are under investigation for use in charge storage applications. These include carbonyl compounds (e. g., quinones[^4^, ^5^]), organic sulfides,^[6]^ conductive polymers (e. g., polythiophenes),^[7]^ N‐oxyl‐radicals,^[8]^ and organometallic coordination compounds.^[9]^ Among the last class of compounds, metal‐containing tetrapyrrole macrocycles (TPMs, i. e. phthalocyanines, porphyrins and related porphyrinoid analogues) have been identified as one of the most promising lead structures due to their unique tuneable redox activity, chemical inertness, and structural stability.[^10^, ^11^, ^12^, ^13^, ^14^, ^15^] Phthalocyanine‐based batteries were already investigated in the past,[^16^, ^17^, ^18^, ^19^, ^20^, ^21^] and have begun to attract renewed attention[^22^, ^23^, ^24^, ^25^] while research into porphyrins[^10^, ^14^, ^26^, ^27^] and porphyrinoid analogues^[15]^ has only recently come into focus.

TPMs are everywhere in nature; heme in hemoglobin or chlorophyll are just two very famous examples to name. Their field of application is growing continuously, particularly in organic photovoltaics,^[28]^ and as therapeutic agents.^[29]^ Porphyrinoids are formed by condensation of pyrrole‐derivatives and aldehydes. The carbonyl‐C of the latter forms the *meso*‐positions connecting the pyrrole rings. Substitution at *meso*‐positions and β‐positions on pyrrole allow diverse modifications of structure and properties. The *trans*‐A2B2 porphyrins used in this study‐ are synthesized via MacDonald condensation[^30^, ^31^] of functionalized dipyrromethene (bearing the A‐group) and another aldehyde (B), followed by metalation and deprotection. This linear multi‐step synthesis inadvertently furnishes low overall yields. However, understanding the basic principles that govern charge storage in TPMs can provide guidelines to devise more readily accessible derivatives that offer superior performance. Indeed, fully symmetrical (A4‐Porphyrins) *meso*‐aryl porphyrins, for example, *meso*‐tetraphenyl porphyrin, TPP^[32]^) can be much more readily synthesized in high yields, even in one‐pot‐reactions combining condensation and metallation^[33]^ and are hence commercially available.

TPMs are quite different from other redox‐active organic compounds^[7]^ in terms of their physical and chemical properties and charge storage mechanism. Porphyrins and phthalocyanines better maintain morphological integrity and structural stability during the charge‐discharge process than quinone‐type organics and conductive polymers. For instance, the performance of quinones with the best balance between high discharge potential and high specific capacity gradually degrades during cycling even with the immobilization of the molecules.^[1]^ On the other hand, the volume expansion/shrinkage during charging‐discharging of conductive polymers induces substantial stress on the mechanical integrity, thus, impairing the cycle life of the electrode.^[7]^ Contrarily, the morphology of porphyrins is retained even after extended cycling,^[34]^ and their long cycle life in metal‐free,^[35]^ and Li‐,[^35^, ^36^, ^37^, ^38^, ^39^] Na‐,[^34^, ^40^, ^41^] K‐,^[42]^ and Mg‐based^[43]^ metal ion storage systems confirms their excellent structural stability. The close intermolecular distances (∼3.5 Å), such as those found in Cu porphyrin π‐stacks,^[36]^ facilitate large spatial overlap between the highest occupied and lowest unoccupied molecular orbitals (HOMOs and LUMOs, respectively) on neighbouring molecules, which strongly affect the electronic properties of the material.^[44]^ The aggregation of such rigid π‐structures (sometimes called self‐assembly^[45]^ or self‐conditioning^[36]^) during the battery operation[^26^, ^42^, ^46^] provides a powerful strategy for suppressing their undesired solubility. These structures are also presumed to enhance the electronic conduction during the charge‐discharge process and to improve retention of the structural integrity upon high rate cycling. Besides, having both p‐type (as of conducting polymers) and n‐type (as of quinone‐type organics) sites, TPMs can accept both cations and anions (b‐type). This leads to a higher capacity (compared to conductive polymers) and higher average discharge potential (compared to quinone‐type organics).[^39^, ^41^, ^47^]

Metal containing TPM electrodes generally show better storage performance than free‐base TPMs.[^34^, ^39^] However, this does not mean that there is no preference for particular applications. To illustrate, mainly Cu^II^‐complexes of phthalocyanines[^39^, ^41^] and porphyrins[^47^, ^48^] and the Ni^II^‐norcorrole complex^[15]^ were utilized as potential cathodes in rechargeable batteries. On the other hand, the augmentation of anode performance with Co^II^‐porphyrin has also been explored.^[37]^ Why are such specific ions preferred over others? Such understanding will provide valuable guidelines for the design of electrode materials, that is, avoiding irreversible formation of species that limit rechargeability and cycling stability. In fact, the role of the metal ion in the whole process is not completely understood yet. As an example, in the case of metal tetraaminephthalocyanine (TAPc), it has been proposed that the Cu preference is dictated by the strong interaction between [CuTAPc]^+/2+^ and PF_6_^−^ and the electropolymerization of the CuTAPc monomers.^[39]^ However, the Cu complex may not be unique in these functions.^[49]^ Besides, the different structures of the complexes with various metal ions and the resulting changes of redox stability window,^[50]^ conductivity^[51]^ and solubility,^[52]^ as well as crystallinity and porosity have been mainly overlooked in designing TPMs‐based electrodes.

**Figure 1:**
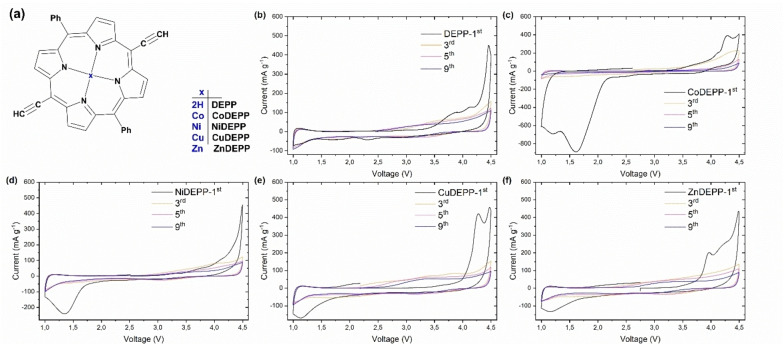
Structures of porphyrin molecules used in this study (a) and cyclic voltammograms for DEPP (b), CoDEPP (c), NiDEPP (d), CuDEPP (e), and ZnDEPP (f), complexes in Li half‐cell with LiPF_6_ electrolyte at the potential range of 1–4.5 V.

Herein, we report a combined experimental and theoretical study of the electrochemical activity across a family of porphyrins with a centrally‐coordinated divalent metal ion of first‐row transition metals from Co to Zn as well as related metal‐free 5,15‐bis(ethynyl)‐10,20‐diphenylporphyrin (DEPP). Figure 1a shows the molecular structure of the compounds used in this study. We are able to demonstrate that the electrochemical performance of porphyrins can be tuned by the choice of metal atom.

## Results and Discussion

The DEPP ligand‐frame was prepared via one‐pot condensation of 3‐(trimethylsilyl)‐propionaldehyde with *meso*‐aryl dipyrromethane in the presence of boron trifluorid‐diethyletherat followed by oxidation with 2,3‐dichloro‐5,6‐dicyano‐1,4‐benzoquinone (DDQ) as an oxidizing agent (Scheme S1). The thereby obtained trimethylsilyl‐ethinyl‐derivate (TMS‐protected trans‐A2B2‐porphyrin) was then either converted into the xDEPP metal complexes (x=Co, Ni, Cu, Zn) followed by deprotection with tetra‐*n*‐butylammonium fluoride (TBAF), or the ligand was directly deprotected to prepare the free ligand 2HDEPP. All compounds were characterized by Nuclear Magnetic Resonance (NMR) Fourier‐Transform Infrared (FTIR), Absorption (UV‐Vis) spectroscopies and Matrix‐assisted laser desorption/ionization‐time of flight (MALDI‐ToF) mass spectrometry, elemental analysis, and powder X‐ray diffraction (XRD). Single crystals suitable for X‐ray diffraction were obtained for the TMS‐protected Co‐complex (CoDEPP‐TMS, see Experimental Section in the Supporting Information and Figures S3–S8).

The variation of metal atoms in DEPPs alters their solubility (Figure S9, Table S1). Density functional theory (DFT) computations were performed to elucidate the effect of the central metal on the electronic properties and molecular geometries of xDEPPs (Figures S10–S12). In brief, the DFT calculations show that the frontier orbitals (FOs) of xDEPPs are mainly distributed on the porphyrin skeleton (Figure S10) while substantial conjugation onto the ethynyl group is also present. However, differences in electron density distribution along the coordinate bonds (x‐N_4_) can be seen, in particular proving the charge transfer between the metal and ligand. Energetically, the HOMO of ZnDEPP is hardly affected with respect to the DEPP reference (Figure S12). This charge transfer is more evident in Ni and Co complexes with vacant d orbitals which further stabilizes the HOMO orbitals (Figure S12a). This is in agreement with the remarkable shorter x‐N bond (around 1.96 Å) in NiDEPP and CoDEPP, than in ZnDEPP and CuDEPP (around 2.0 Å) (calculated and experimental data in Table S2). On the other hand, a partial shift of the LUMO toward the central metal atom is noticeable when going from Zn to Cu, Ni, and Co. As a result, the overall energy gap between HOMO and LUMO is in the order of CoDEPP>NiDEPP>CuDEPP>ZnDEPP (Figure S12b). Consequently, we expect an enhanced electron transfer for CuDEPP and ZnDEPP. In contrast, Co and Ni could use their easily available higher HOMO‐1 orbitals for further redox reactions (Figure S12c).

The electrochemical performances of the xDEPP complexes were evaluated in half cells against a lithium metal anode. To minimize the partial loss of xDEPP from the cathodes by dissolution into the electrolyte, the volume of electrolyte used was restricted to around 25 μL mg^−1^ of xDEPP in all cells. Figure 1b–e compare the cyclic voltammograms of xDEPP complexes within the voltage window of 1.0–4.5 V and clarify the impact of the central metal on the reversibility of redox reactions. Furthermore, the discharge capacities and CE for all of the complexes in the initial 10 cycles are shown in Figure S13b. In the first anodic sweep, distinct irreversible oxidative peaks appeared above 3.8 V which seem to be specific for this class of compounds and are most likely associated with the self‐conditioning feature of the xDEPPs as evidenced by spectroscopic studies^[36]^ and also observed in the Na‐,^[34]^ K‐^[35]^ and Mg‐^[43]^ based systems and will not be discussed further here.

All four metal complexes exhibit prominent irreversible reductive peaks with onsets below 2.2 V, which only occur in the first cycle (Figures 1 and S13a). Notably, the potential of the peaks consecutively shift in the order of the elements as follows: ZnDEPP and CuDEPP 1.12 V; NiDEPP 1.35 V; CoDEPP 1.61 and 1.20 V. If electrons were added to the π‐ring system (as of DEPP) only, such variations could barely be expected. Instead, such irreversible peaks are probably related to the further reductions occurring in the central metal rather than the π‐ring system. Indeed, electron uptake by a metal‐center has been confirmed in Co^II^‐porphyrin,^[53]^ Ni^II^ complex,^[54]^ and Cu^II^‐porphyrin.^[36]^ The position and the intensity of the peaks for CoDEPP are sharply different from that of other metal porphyrins (Figure S13a). Additionally, the Co complex reveals distinct capacity loss in the initial 10 cycles (Figure S13b). DFT calculations suggest that the unoccupied orbitals have more metal character in CoDEPP than the other metal porphyrins (Figure S10). Moreover, two peaks are apparent for CoDEPP, signifying a difference in the nature of the reductive process in CoDEPP and suggesting that the successive electron is also being added to the central metal here. Indeed, the preference for electron uptake by a Co^II^ metal‐center has been reported in other Co^II^‐porphyrin complexes.[^53^, ^55^]

**Figure 2:**
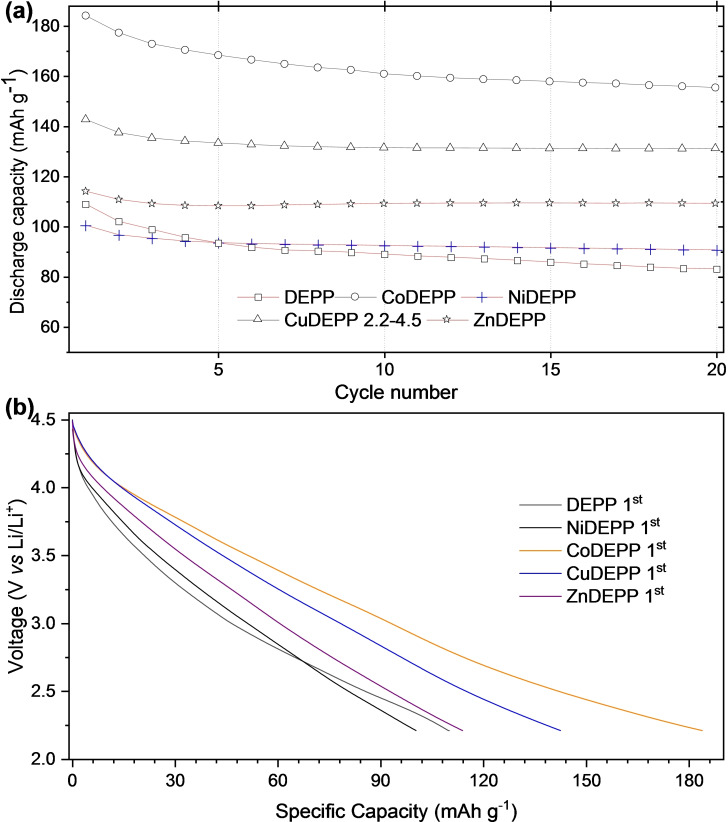
Cycling performance (a) and first discharge curves (b) of xDEPPs at 100 mA g^−1^ in the potential range of 2.2–4.5 V.

Based on the CV results and to get a detailed understanding of the intrinsic material properties, the cells were cycled between the voltage limits of 4.5 and 2.2 V at 100 mAh g^−1^ (Figure 2). After the first charging process, more reversible discharge‐charge behaviors were observed and the coulombic efficiency gradually increased during the initial cycles and remained close to 90 % during 10 successive cycles for all xDEPPs (Figures S14–S18).

The discharge capacity of the xDEPP electrodes follows the order of CoDEPP>CuDEPP>ZnDEPP>DEPP>NiDEPP with first discharge capacities of 184, 143, 114, 109, and 101 mAh g^−1^, respectively. These values correspond to specific energy densities of 565, 446, 356, 327, and 307 Wh kg^−1^ for electrodes with Co‐, Cu‐, Zn‐, metal‐free‐, and Ni‐complex, respectively. The lack of evident voltage plateaus in the discharge profiles (Figure 2b) implies rapid pseudocapacitive redox reactions. Such sloped discharge profiles can be attributed to the combination of double‐layer capacitance and multiple redox reactions,^[56]^ and a similar profile has often been observed in other organic electrodes.[^15^, ^56^, ^57^, ^58^] The significantly lower capacity of NiDEPP could be due to the fact that Ni^2+^ is too small to perfectly fit into the square planar cavity formed by the four pyrrole nitrogen atoms (ionic radii Co^2+^≅Cu^2+^≅Zn^2+^>Ni^2+^). This will result in less efficient intermolecular interactions and higher solubility of NiDEPP in the electrolyte solution (Table S1). Interestingly, Ni^2+^ fits perfectly into the smaller cavity of TPMs, for example, in norcorrole.^[59]^ This prevents the undesired solubility and results in meaningfully improved reversible capacity for the Ni complex.^[15]^

**Figure 3:**
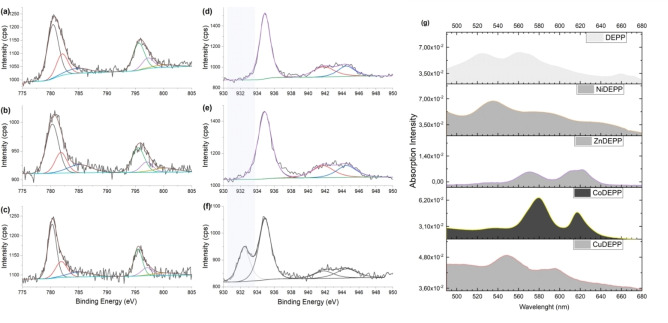
XPS measurements in the Co 2p region for CoDEPP (a, b, c) and the Cu 2p region for CuDEPP (d, e, f) and CuDEPP (d, e, f) electrodes at open‐circuit voltage (OCV) (a, d), charged (b, e), and discharged (c, f) states. UV/Vis absorption spectra of xDEPPs (Q‐band region between 480–680 nm) (g).

For CuDEPP, the discharge capacity of 138 mAh g^−1^ in the second cycle is close to the theoretical value of 140 mAh g^−1^ expected for the three‐electron reaction [CuDEPP]^−^↔[CuDEPP]^2+^. The added capacity attained in CoDEPP corresponds to an additional 1 e^−^ redox and a capacity of around 45 mAh g^−1^. The redox activity of the central atoms was investigated by X‐ray photoelectron spectroscopy. While the measurements indicated in the case of CuDEPP no change of the Cu oxidation state (+II) when going from the pristine to the charged state, reduction from Cu(+II) to Cu(+I) of a part of the Cu center atoms is noticed upon discharge (Figure 3d–f). Indeed, the formation of mixed‐valence metal‐porphyrins[^60^, ^61^] and, in particular, Cu^2+^/Cu^+^ species in Cu‐porphyrins in the redox process was reported earlier.[^36^, ^62^] In the case of CoDEPP, the measurements did not show any indication for a change of the Co oxidation state (which stays +II) during cycling (Figure 3a–c). Although the oxidation of Co^2+^ to Co^3+^ could theoretically provide an additional capacity of 47 mAh g^−1^, the results of XPS measurements discard the possibility of the involvement of metal redox activity. It suggests that the third electron is also being extracted from the ring in the Co‐complex to produce [Co(+II)DEPP]^3+^ This view is also supported by the results of our theoretical investigation of the frontier orbitals, which indicates the smallest energy variance between HOMO and HOMO+1 orbital for CoDEPP than other xDEPPs (Figure S12c).

**Figure 4:**
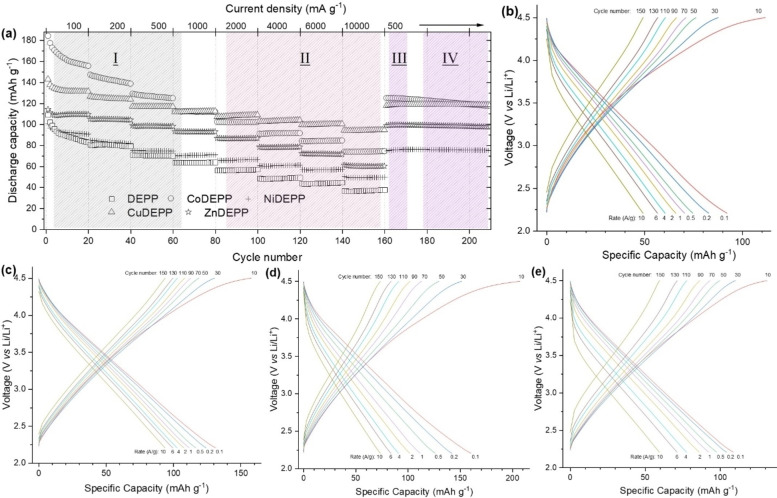
Rate performance of the xDEPPs (a) and selected voltage profile of NiDEPP (b), CuDEPP (c), CoDEPP (d), and ZnDEPP (e) at various current densities from 0.1 to 10 A g^−1^ in the potential range of 2.2–4.5 V (details in the Supporting Information; Figure S14–S18).

The comparison of the results of rate performance tests for electrodes with different xDEPPs in Figure 4 (details in Figures S14–S18) shows several interesting features. First of all, the introduction of the metal atoms obviously enhances the discharge capacity and the rate capability of DEPP compared to the free base at different rates ranging from 0.1 to 10 A g^−1^. In general, specific metal centers can directly take part in the redox processes or the unpaired electrons in some metals/states may also catalyze ion insertion and extraction[^19^, ^20^] which lends additional capacity and redox stability and/or facilitates the redox reactions. In the lower current rates (up to 1000 mA g^−1^, region I), the discharge capacities of the xDEPP electrodes show a clear trend with CoDEPP>CuDEPP>ZnDEPP>NiDEPP>DEPP. At higher current rates (from 2000 to 10000 mA g^−1^, region II), however, CuDEPP shows the highest discharge capacity. The rate capability factor (here defined as the discharge capacity at 10000 mA g^−1^ divided by the value at 100 mA g^−1^) is 71.9, 54.9, 54.0 46.3, and 41.6 % for CuDEPP, ZnDEPP, NiDEPP, CoDEPP, and DEPP, respectively. In all cases, the original capacities at a rate of 500 mAh g^−1^ were fully recovered after operation at higher rates (region III).

**Figure 5:**
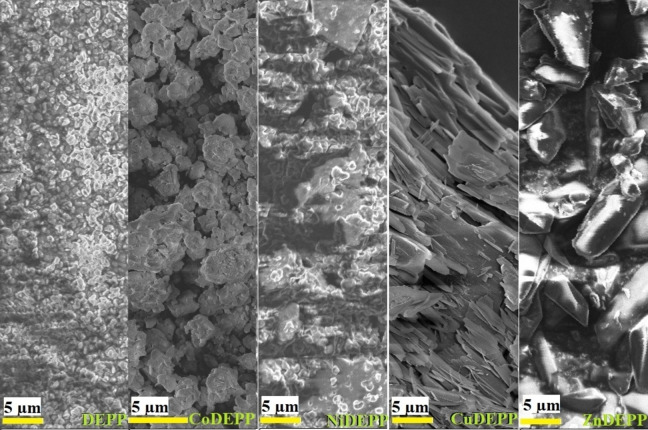
SEM images of the different xDEPP electrode materials used in this study.

**Figure 6:**
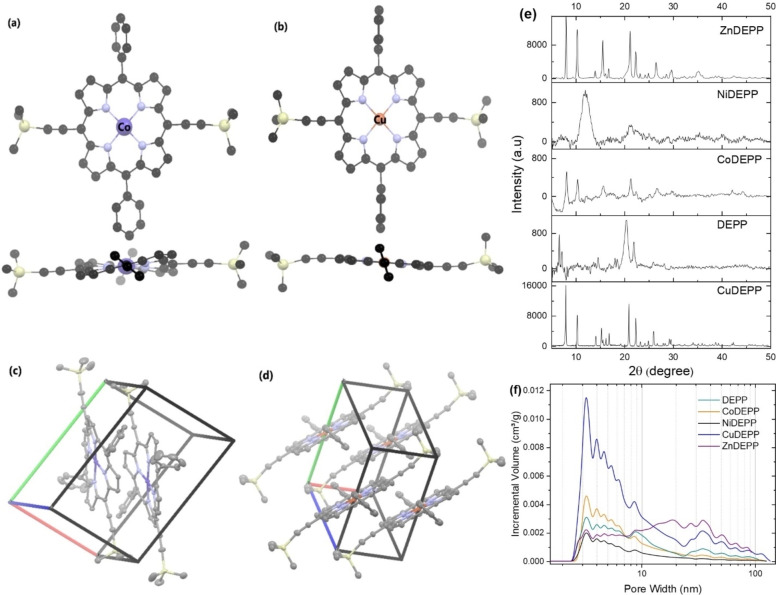
Top and side view of the (a) ruffled Co‐porphyrin and (b) planar Cu‐porphyrin, and packing of the (c) Co‐complex and (d) Cu‐complex in the crystal structure of CoDEPP‐TMS and CuDEPP‐TMS. Images are generated from CCDC No. 1506859^[36]^ and 2143452. Powder XRD patterns (e) and pore size distribution curves (f) of the xDEPP active materials used in this study.

The electrodes exhibit much‐varied rate capabilities depending on the nature of the central metal. In general, the acceleration of ion diffusion or the enhancement of electronic conductivity in composite electrodes improves the rate capability. Even though the type of the central metal and the nature of π‐bond conjugation and resulting stacking are expected to influence the electrical conductivity of porphyrins,^[63]^ having a high amount of conductive carbon in the electrode minimizes the influence of the central metal on the electrical conductivity of the electrode. The observed rate capability trend of the different xDEPPs was most likely caused by differences in the bulk structure since both Cu and Zn containing porphyrins (with higher rate capability factor) turned out to be more crystalline as derived from SEM (Figure 5) and powder XRD (Figure 6e) results. A comparison of powder XRD patterns of the bulk xDEPP‐materials investigated herein showed that only ZnDEPP and CuDEPP could be obtained as highly crystalline solids (Figure 6), while NiDEPP and CoDEPP were found to be largely amorphous. Similar shapes are also retained after extended cycling (Figure S19). For CuDEPP, a uniform needle‐like morphology was observed, where the length of the needle is in the order of a few 10 μm. ZnDEPP shows a different morphology with crystallites of 5–10 μm in size that aggregate in random orientation, which possibly induces a less preferred orientation.

The degree of molecular ordering and packing can impact various solid‐state properties of TPMs, including their charge transport properties.[^64^, ^65^] DFT calculations revealed that the molecular geometry is significantly affected by the nature of the metal. While most xDEPPs exhibit a nearly planar structure in their DFT‐optimized geometries, NiDEPP and CoDEPP appear to undergo certain saddling distortions (Figure S20). Such varied geometries will result in an altered conjugation pathway, affecting their UV/Vis absorption spectrum.[^66^, ^67^, ^68^] The UV/Vis spectrum of DEPP (Figure S21) consists of a strong transition (S0→S2, Soret band) and four weak transitions (S0→S1, Q‐bands). While introducing the central metal causes only minor changes to the Soret band, it usually strongly affects the wavelengths and intensities of the Q‐bands. Generally, the number of Q‐bands is reduced upon coordination to metals due to the higher symmetry of the porphyrin‐metal complex. Besides, it is expected that such distortion in metalloporphyrins results in a spectral redshift.[^69^, ^70^, ^71^, ^72^, ^73^] Considering Figure 3g, with the insertion of Ni in DEPP, nonplanar distortions bring about mild redshifts in the electronic spectra of NiDEPP (more than 10 nm in Q‐bands). With the insertion of Co, a significant redshift was observed for the main Q‐bands, from the initial ∼525 and 560 nm in DEPP to ∼580 and 620 nm in CoDEPP with an increase in the relative absorption intensity of the bands. The results obtained are in excellent agreement with DFT calculations. Non‐planarity generally increases the solubility of condensed aromatic systems.[^74^, ^75^] This might explain the successively increased solubility (Figure S9, Tables S1 and S3), which is suggested to be a reason for the notable decline in the discharge capacity of the Co‐ and Ni‐complexes in the initial cycles.

A molecular structure of the CoDEPP‐TMS was obtained experimentally by single crystal X‐ray diffraction (Figure 6, see Table S4 for details), and could be compared to the previously reported structure of TMS‐protected Cu‐complex (CuDEPP‐TMS).^[36]^ Although single‐crystal complexes are obtained from the protected complexes, the core porphyrin structure and packing seem not to be affected significantly, and the structures are in good agreement with the DFT‐optimized structures of CoDEPP and CuDEPP (Figure S20). CuDEPP crystallizes in the triclinic space group *P* $\bar{1}$ , with one molecule in the unit cell (*Z*=1) and cell length *a*=6.068, *b*=11.958, *c*=12.765, and angles of *α*=79.1°, *β*=81.1°, *γ*=84.9°.^[36]^ Within a layer, the CuDEPP molecules are partially tilted so that molecular planes do not overlap, and a Cu atom on one molecule sits adjacent to two ethynyl groups on neighboring molecules of the same layer. Besides, layers are stacked so that aromatic porphyrin cores are not directly lying above each other, while the phenyl rings remain twisted out of the porphyrin core plane.

The CoDEPP molecules also crystallized in the triclinic space group *P* $\bar{1}$ , with *Z*=2 and cell length *a*=9.368, *b*=14.055, *c*=15.002, and angles of *α*=66.8°, *β*=82.4°, *γ*=79.8°. In contrast to the Cu‐complex, the porphyrin core in CoDEPP^TMS^ CoDEPP‐TMS is not planar but rather assumes a saddled geometry with N−Co−N angles of 173.3° and −173.3° (compared to 180° for CuDEPP‐TMS). The torsion between N atoms and pyrrolic C atoms is more than 3° in the Co‐complex (compared to less than 1.5° for the Cu‐complex). In addition, there are apparent differences in torsion angles between the plane determined by the pyrrole rings and the plane of the *meso*‐substituted phenyls (Figure S22). The dihedral angle between the aryl group and the porphyrin plane in CuDEPP is around 69° with coplanar aryl groups enabling effective packing. In CoDEPP, the π‐system is saddled significantly so that both phenyl rings are not coplanar, as they do appear in CuDEPP (dihedral angles of 75° and 57°). The stacking of the metal porphyrin layers in the case of CuDEPP (Figure 6d) is expected to significantly increase the π‐electron overlap and reduce the HOMO and LUMO gap. For instance, the bandgap for the single CuDEPP molecule is 2.51 eV, whereas it decreases to 2.3 eV for a stack of 3 molecules (Figure S23).

**Figure 7:**
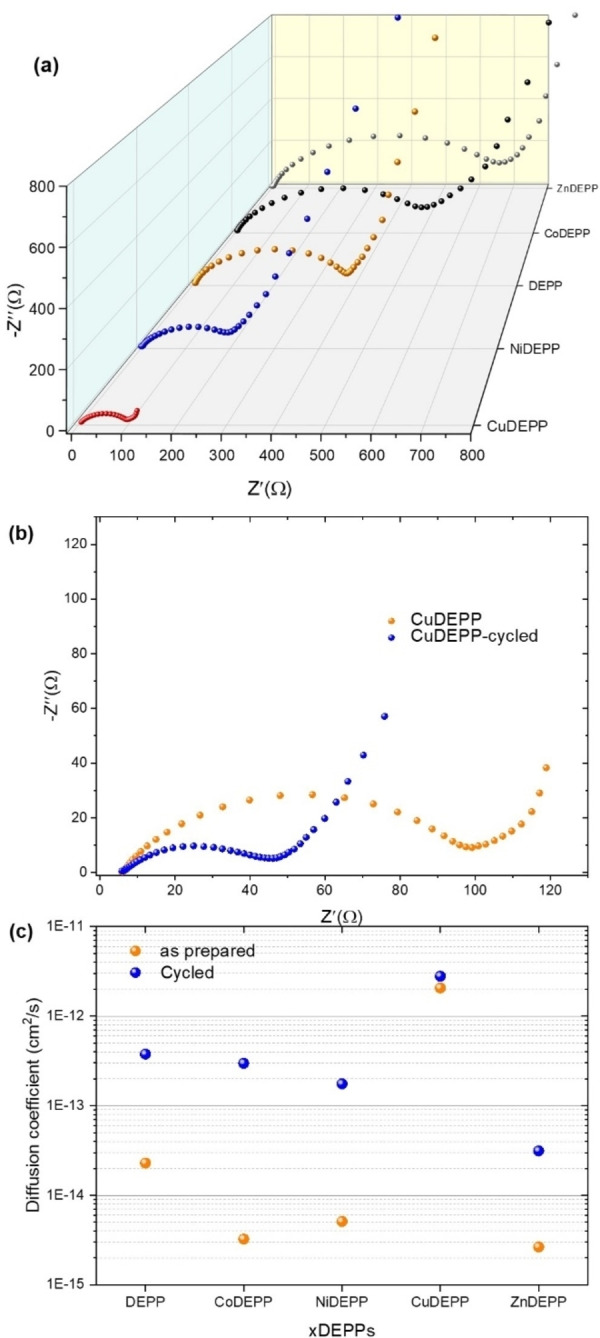
Comparison of Nyquist plots of the xDEPP electrodes before cycling (a) and CuDEPP electrodes before cycling and after the 10th cycle (b). Estimated diffusion coefficient in fresh and cycled xDEPPs determined by EIS (c). (The equivalent circuit model and the fitting results are presented in Figure S24 and Table S5.)

To gain further insight into the electrochemical properties of the xDEPPs, electrochemical impedance spectroscopy (EIS) studies were conducted (Figure 7a, see also Figure S24). The data were fitted with an equivalent circuit (Figure S24 and Table S5), in which *R*_s_ represents the internal resistance of the cell, *R*_ct_ denotes the charge transfer resistance which refers to the resistance of electrons and ions, CPE is associated with the constant phase element, and *W*_o_ is the Warburg impedance representing the diffusion of ions within the electrode. Figure 7a depicts a close‐up view of the high‐frequency region of Figure S24. According to the fit, the *R*_ct_ values were found to be 90.6, 167.4, 361.9, 452.4, and 608.1 Ω for CuDEPP, NiDEPP, DEPP, CoDEPP, and ZnDEPP, respectively. These data explain the improved rate performance of the CuDEPP electrode. Remarkably, after 10 cycles, the *R*_ct_ of xDEPPs dramatically reduces to 38.6, 42.0, 38.6, 51.3, and 86.3 Ω for CuDEPP, NiDEPP, DEPP, CoDEPP, and ZnDEPP, respectively. This demonstrates the facilitated charge transfer and improved kinetics of all xDEPPs after the conditioning in the initial cycles.^[34]^ Further evaluation revealed that the ion diffusion coefficient of CuDEPP is by 2–3 orders of magnitude higher than that of other xDEPPs (Figure 7c). In general, lithium‐ion diffusion kinetics correlate with the porosity and specific surface area, as increased contact areas between active material and electrolyte provide more diffusion channels for ions.^[76]^

The surface area of the xDEPPs was obtained from the N_2_ adsorption–desorption isotherm experiments (Figure S25). Aside from the higher overall surface in CuDEPP, the pore size distribution curves (Figure 6f) indicate higher pore volume for pores having sizes above a few nm. Such large pores are ideal for fast redox reactions to provide fast mass‐transport of the electrolyte.^[77]^ Specifically, a high fraction of the pores with sizes of 1.5 – 3 nm was found in CuDEPP. These pore sizes were found to be suitable for anion insertion and could match well with the size of solvated PF_6_^−^ ions,^[78]^ resulting in outstanding rate capability of CuDEPP retaining 72 % of its capacity at 10000 mA g^−1^. At a molecular level, both CuDEPP and CoDEPP crystalize in triclinic space groups and adopt layered structures that give rise to two‐dimensional diffusion paths perpendicular to the planes of the coplanar π‐stacked porphyrin cores. However, while the ring system of CuDEPP is nearly perfectly planar, CoDEPP adopts a saddled geometry. Besides, CuDEPP molecules also exhibit less π‐overlap, which is evidenced for example by larger distances between Cu atoms within the same stack (*d*(Cu⋅⋅⋅Cu)=(6.069(1) Å) than between Co atoms (*d*(Co⋅⋅⋅Co)=5.436 Å and 5.272(1) Å). A consequence of the “flatter” π‐stacking is less steric interference of *meso*‐phenyl rings in adjacent CuDEPP molecules, which leaves more spaces in between the sheeted π‐stacks and gives rise to the well‐defined pore structure in CuDEPP (Figure S26). This could also ease ion insertion/extraction into/from the bulk material.

**Figure 8:**
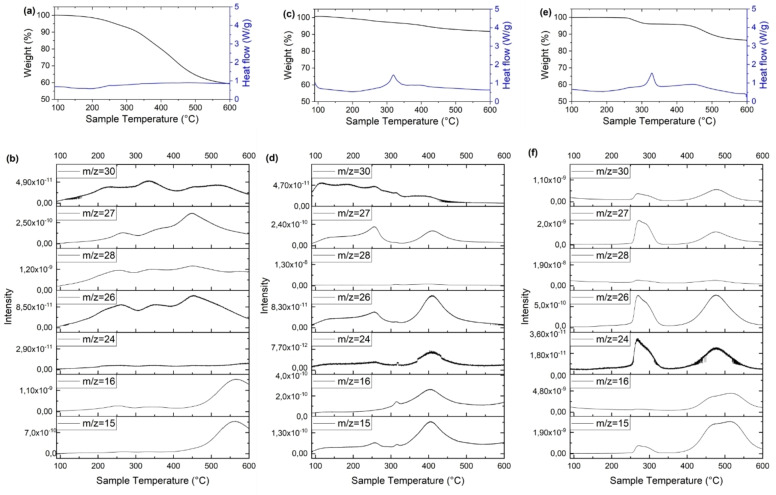
Thermogravimetric analysis, differential scanning calorimetry and mass spectrometry of selected xDEPPs. The TGA and DSC profiles (a, c, e) and analysis of the evolved gases (b, d, f) of DEPP, CoDEPP and CuDEPP, respectively.

The thermal properties of xDEPPs were studied by thermogravimetric analysis and differential scanning calorimetry (TGA‐DSC). The results showed that decomposition of DEPP starts at above 200 °C with a 5 % weight loss at a temperature (*T*_(5)_) of around 270 °C (Table S6) and no prior phase transition (Figure 8). Except for the Ni complex, introducing the central metal improves the thermal stability significantly, with the *T*_(5)_ rising to above 400 °C (Table S6). Interestingly, Co and Cu complexes retained around 85 wt % at 800 °C. Such high thermal stability is a critical feature to ensure safe operation of the storage system and its possible operation at elevated temperatures.^[79]^ These thermal stabilities are equal to or exceed those of most organic electrode materials such as the anthraquinone‐[^80^, ^81^] terephthalate‐^[82]^ phosphazene‐^[83]^ and polyimide‐based^[84]^ materials. Furthermore, TPMs are oxygen‐free cathodes, which eliminates the possibility of oxygen gas evolution from active material decomposition. This stands in contrast to commercial Li‐ion batteries (LIBs) featuring layered transition metal oxides as cathode materials. Here, exothermic decomposition of the cathode at elevated temperatures is the primary safety concern, as it can result in instantaneous oxygen release leading to further increase in temperature and pressure, which can end with fire and even explosion.^[85]^ The oxygen‐free structures are generally known to be thermally safe, avoiding a common battery thermal runaway pathway that requires O_2_ liberation.^[86]^

According to the TGA profiles (Figure 8), both Cu and Co complexes are highly stable up to 260 °C. Before the first degradation region in the Cu complex, no gas release was also observed (the minor release in the Co complex could be due to trace impurities). The analysis of the evolved gases with mass spectrometry revealed that besides general C_*x*_H_*y*_ species (*m*/*z*: 15, 16, 24, 26, 28), hydrogen cyanide (HCN, *m*/*z* 27) was identified at 275 °C (after 2 wt % loss in TGA) and 470 °C. The formation of highly toxic hydrogen cyanide poses an additional risk for this type of material. However, compared to the overall organic content of the battery, the nitrogen content and by correlation, the HCN concentration‐ is small and the material decomposition temperature (2 % weight loss at 275 °C for the CuDEPP) is higher than the boiling point of electrolyte solvents, for example of propylene carbonate (242 °C). Therefore, the risk from the general LIB electrolyte decomposition is substantially higher than the risk from active material decomposition.

## Conclusion

In this study, a straightforward procedure was used to prepare A2B2‐type porphyrin DEPP and the corresponding first‐row transition metal complexes from Co to Zn. A comparative study of the bulk structures and electrochemical properties of xDEPP showed that the choice of the central metal atom may not only facilitate noncovalent π–π interactions, which result in a stable battery operation, but also have significant consequences on the capacity and rate capability of the resulting energy storage systems. For the first time, we report that the choice of the central metal results in extra redox reactions in the π‐ring system. These findings open new opportunities to design high‐capacity cathodes for advanced Li‐ and post‐Li‐ion storage systems. Additionally, it seems that the ion diffusion coefficient in xDEPPs is the product of porosity and specific surface area, whereas the rate capability is the product of the crystallinity. Therefore, appropriate control of these factors can considerably improve the performance of TPM electrodes for specific applications. We expect to see considerable optimization of metal‐TPMs by using the different central metal atoms and altering their size and morphology to stabilize the structure further and increase their rate capability and cycling stability.

## Experimental Section

### Methods

Nitrogen adsorption measurements were performed to determine the BET specific surface area and pore characteristics. The measurements were performed with a ASAP 2020 surface area and porosity analyzer apparatus by Micromeritics Instrument Corporation. Before measurements, the powder samples were dried and degassed at 100 °C overnight until a pressure lower than 0.15 mbar was reached. The specific surface area and the micropore area were calculated with the standard instrument software in accordance with the BET method.

Electrochemical impedance spectroscopy (EIS) was used to investigate the charge transfer resistance and ionic diffusion behavior of the developed xDEPP cathodes. Due to the partial dissolution of xDEPPs over prolonged measurement periods, EIS was preferred over cyclic voltammetry. EIS measurements in the frequency range of 1 MHz to 50 mHz were performed on a VMP3 multichannel potentiostat from BioLogic. The spectra were fitted using EC‐Lab software and used to calculate the ion diffusion coefficient according to the following Equation 1:^[87]^

(1) $$D=\frac{R^{2}T^{2}}{{2A}^{2}n^{4}F^{4}C^{2}\sigma^{2}}$$

where *D* is the diffusion coefficient [cm^2^ s^−1^], *R* is the gas constant (8.314 J mol^−1^ K^−1^), *T* is the absolute temperature (294.3 K), *A* is the surface area of the cathode (1 cm^2^), *n* is the number of transferred electrons, *F* is the Faraday constant (96485 C mol^−1^), *C* is the concentration of electrolyte (3×10^−3^ mol cm^−3^ ), and *σ* is the Warburg factor [Ω s^−1/2^] and is determined by fitting to an equivalent circuit model which includes a Warburg impedance.

The ^1^H NMR measurements were carried out with a Bruker DRX‐500 at 500 MHz using deuterated chloroform (CDCl_3_) solvent. The measurements by Fulmer et al.^[88]^ served as a reference. The coupling constants are given in Hz. The chemical shift is given in ppm and is based on tetramethylsilane as the internal standard. The multiplets are shown in abbreviated form (s: singlet, d: doublet, t: triplet, q: quartet and m: multiplet). The indication of the chemical shift relates to the signal center; except for multiplets for which the entire range is indicated. The evaluation was carried out with the TopSpin 4.0.6 software from Bruker.

The IR spectra were recorded with the Magna FTIR 750 spectrometer from Nicolet. The substance to be examined was prepared as a KBr pellet. The evaluation was carried out with the “OPUS 7.0” program. The vibration numbers were given in wavenumbers of the unit cm^−1^. Measurements were done in a range of 400–4000 cm^−1^. The vibrations were assigned with the help of literature (Abbreviations: def: deformation; sh: sharp; str: stretching; sym: symmetric; vib: vibration).^[89]^ The MALDI‐ToF mass spectra were recorded with the “WATERS Synapt” device from WATERS. No matrix was used. The information is given by relative masses [*m*/*z*] and relative intensities [%] of the strongest signal in each case. The UV/Vis measurements were taken with a Cary 500 Scan (Varian) in dichloromethane (≤5×10^−5^ M). X‐ray diffraction (XRD) patterns of the powders were recorded using a Bruker D8 diffractometer with Bragg‐Brentano geometry using Cu−Kα radiation. The SEM images were taken by a Zeiss Leo Gemini 1530 System. Elemental analyses were carried out using a vario micro cube in CHNS‐mode. The chemical state of the elements was determined by X‐ray photoelectron spectroscopy (XPS) using a Specs XPS system with monochromatized Al Ka radiation and a Phoibos 150 energy analyzer. The samples were kept under Ar atmosphere during transfer to the XPS system. For binding energy calibration, the main C 1s peak was set to 284.8 eV. Peak fitting was done with Casa XPS using Shirley‐type backgrounds and Gaussian‐Lorentzian peak profiles. Simultaneous thermos‐gravimetric analysis, differential scanning calorimetry and mass spectrometry (TGA‐DSC‐MS) were performed with a Setaram thermal analyzer SENSYS evo TGA‐DSC equipped with a Pfeiffer OmniStar mass spectrometer to analyze the evolved gas. The analyses were performed under an argon atmosphere at a heating rate of 10 °C min^−1^.

Deposition Number 2143452 (for CoDEPP‐TMS) contains the supplementary crystallographic data for this paper. These data are provided free of charge by the joint Cambridge Crystallographic Data Centre and Fachinformationszentrum Karlsruhe Access Structures service.

### Electrode preparation

The xDEPP electrodes were prepared via a water‐based process, with 32 wt % solid content. Sodium carboxymethyl cellulose (CMC) and styrene‐butadiene rubber (SBR) were utilized in the process with a mass ratio of 1 : 1.1. The CMC solution with the desired concentration was prepared by dissolving CMC in distilled water under heating and stirring overnight. The electrode slurries were prepared by mixing the xDEPP (46.4 wt %), Super P (46.4 wt %), and binder (7.2 wt %) in distilled water using a Thinky mixer. A homogeneous slurry was obtained after stirring at 2000 rpm for 10 min. The obtained slurry was then coated on a carbon‐coated aluminum foil with a doctor blade. Finally, the dried coating was punched into 11.8 mm discs which were further dried at 70 °C in a vacuum for 15 h. The loading of active materials is between 1 and 1.3 mg cm^−2^.

### Computational

All calculations on xDEPPs were performed with the Gaussian 16 program packages. Geometry optimizations were performed in the gas phase using the B3LYP functional of the DFT method. Hydrogen, carbon, and nitrogen atoms were treated with the 6‐31G (d,p) basis set, while metal atoms were treated with the LANL2DZ basis set.

### Synthesis

The synthesis of A2B2‐porphyrins is straightforward and consists of three synthesis steps (Scheme S1). In step one, we synthesize the appropriate *meso*‐dipyrromethane from pyrrole and an aldehyde with the intended substituent with the electron‐withdrawing group. In step two, the ring‐closing reaction takes place, flowing the Macdonald condensation. The condensation takes place between the *meso*‐dipyrromethane and (trimethylsilyl)‐propiolaldehyde. The metalation of the developed porphyrin is straightforward and conditions depend on the used metal:

#### [5,15‐bis(trimethylsilylethynyl)‐10,20‐diphenylporphinato]cobalt(II) (CoDEPP‐TMS) (4)

The free‐base porphyrin (**2**) (0.052 g, 0.08 mmol) was dissolved in 10 mL chloroform and 10 mL acetic acid, Co(OAc)_2_ ⋅ 4 H_2_O (0.2 g, 0.83 mmol) was added and the mixture was refluxed for 4 h. The solvent was removed under vacuum. After column chromatography (Al_2_O_3_, 10 % dichloromethane (DCM) in hexane→100 % DCM) a blue solid was obtained with a yield of 85 % (0.048 g). ^1^H NMR (paramagnetic compound); MALDI‐ToF‐MS: calculated for C_42_H_36_CoN_4_Si_2_ [M]^+^: *m*/*z*: 711.2; found 711.0 (100 %); UV/Vis (CHCl_3_): 428, 554, 589 nm; IR (KBr): $\tilde{\nu }$ =3436.01, 2924.02, 2142.49 (C≡C), 1623.01, 1347.46, 1246.49 (Si(CH_3_)_3_ sh sym. CH_3_ def vib), 1210.95, 1167.12, 1068.64, 1004.21, 845.83 (Si(CH_3_)_3_ rocking vib), 796.00 (‐Ph out of plane def vib), 752.87, 705.33 cm^−1^ (ring out of plane def vib).

#### [5,15‐bis(trimethylsilylethynyl)‐10,20‐diphenylporphinato]nickel(II) (NiDEPP‐TMS) (5)

The free‐base porphyrin (**2**) (0.1 g, 0.15 mmol) and the Ni(OAc)_2_ ⋅ 4 H_2_O (0.35 g, 1.4 mmol) were dissolved in 67 mL chloroform and 33 mL methanol. The reaction mixture was heated under reflux for 3 d. The mixture was allowed to cool to room temperature, and the solvent was removed under a vacuum. The crude product was dissolved in chloroform and extracted two times with water. The collected organic phase was dried over sodium sulphate. Chloroform was removed under vacuum. The product was dissolved in THF and water was added, THF was removed under vacuum. The precipitated product, a blue solid, was filtrated and obtained with a yield of 23 % (0.025 g). ^1^H NMR (paramagnetic compound). MALDI‐ToF‐MS: calculated for C_42_H_36_N_4_NiSi_2_ [M]^+^: *m*/*z*: 710.18; found 711.14 (100 %) [M‐H]^+^; UV/Vis (CHCl_3_): 431, 552, 598 nm; IR (KBr): $\tilde{\nu }$ =3432.21, 2957.59, 2144.88 (C≡C), 1627.39, 1348.99, 1247.29 (Si(CH_3_)_3_ sh sym. CH_3_ def vib), 1210.95, 1167.12, 1072.75, 1004.78, 844.93 (Si(CH_3_)_3_ rocking vib), 795.75 (−Ph out of plane def vib), 752.87, 705.47 cm^−1^ (ring out of plane def vib); Elemental analysis calcd. for C_42_H_36_NiN_4_Si_2_: C 70.89 H 5.10 N 7.87, found: C 67.31 H 5.11 N 7.22.

#### General procedure for deprotection of TMS‐group for CoDEPP (9) and NiDEPP (10)^[90]^

The porphyrin (0.05 mmol) was dissolved in 20 mL dry THF and 1 mL of 1 M solution of TBAF in THF was added. The mixture was stirred overnight under an argon atmosphere. The reaction was quenched by adding 50 mL of water. THF was removed under reduced pressure. The precipitate was filtrated and dried overnight at 100 °C and 2.0 ⋅ 10^−2^ mbar.

#### [5,15‐bis(ethynyl)‐10,20‐diphenylporphinato]cobalt(II) (CoDEPP) (9)

^1^H NMR (paramagnetic compound); MALDI‐ToF‐MS: calculated for C_36_H_20_CoN_4_ [M]^+^; *m*/*z*: 568.1; found 567.1 (100 %) [M−H]^+^; UV/Vis (CHCl_3_): 434, 580, 617 nm; IR (KBr): $\tilde{\nu }$ =3430.21, 3264.38 (−C≡C−H−CH str.), 2914.28, 2857.14, 2099.69, 1596.71, 1544.10 (C=C/N in plane vib), 1458.16, 1349.96 (−C≡C−H−CH wagging vib overtone), 1206.57, 1074.03, 1005.66, 795.83 (−Ph out of plane def vib) 752.98, 704.18 (−Ph ring out of plane def vib), 667.39, 619.08 cm^−1^; elemental analysis calcd. for C_36_H_20_CoN_4_: C 76.19 H 3.55 N 9.87, found: C 71.56 H 4.79 N 7.56; yield 97 % (27.52 mg).

#### [5,15‐bis(ethynyl)‐10,20‐diphenylporphinato]nickel(II) (NiDEPP) (10)

MALDI‐ToF‐MS: calculated for C_35_H_21_NiLiN_4_ [(M+3H^+^‐CH_3_)^3−^+H^+^+Li^+^]^−^; *m*/*z*: 561.1; found 561.1 (100 %) [(M+3H^+^‐CH_3_)^3−^+H^+^+Li^+^]^−^; IR (KBr): $\tilde{\nu }$ =3442.80 (−C≡C−H−CH str.), 2958.69, 1566.02 (C=C/N in plane vib), 1484.93, 1352.43 (−C≡C−H−CH wagging vib overtone), 1206.57, 1160.83, 1072.00, 1004.93, 795.25 83 (−Ph out of plane def vib), 753.25, 701.85 cm^−1^ (−Ph ring out of plane def vib); UV/Vis (CH_2_Cl_2_): 421, 535, 572, 639 nm; elemental analysis calcd. for C_36_H_20_NiN_4_: C 76.22 H 3.55 N 9.88, found: C 72.06 H 4.93 N 7.88; Yield 69 % (19.57 mg).

## Conflict of interest

The authors declare no conflict of interest.

1

## Supporting information

As a service to our authors and readers, this journal provides supporting information supplied by the authors. Such materials are peer reviewed and may be re‐organized for online delivery, but are not copy‐edited or typeset. Technical support issues arising from supporting information (other than missing files) should be addressed to the authors.

Supporting Information

## Data Availability

The data that support the findings of this study are available in the supplementary material of this article.
