# Supplementary material for: Molecular Engineering of Metalloporphyrins for High‐Performance Energy Storage: Central Metal Matters
Source: ChemSusChem. 2023 Jan 13;16(3):e202202090. doi: 10.1002/cssc.202202090 (PMC10107660; doi:10.1002/cssc.202202090)
Supplement: Supplementary file 1 — Supporting Information [file CSSC-16-0-s001.pdf]

ChemSusChem

Supporting Information

# Molecular Engineering of Metalloporphyrins for High-Performance Energy Storage: Central Metal Matters

Shirin Shakouri, Ebrahim Abouzari-Lotf,* Jie Chen, Thomas Diemant, Svetlana Klyatskaya, Frank Dieter Pammer, Asato Mizuno, Maximilian Fichtner,* and Mario Ruben*© 2022 The Authors. ChemSusChem published by Wiley-VCH GmbH. This is an open access article under the terms of the Creative Commons Attribution License, which permits use, distribution and reproduction in any medium, provided the original work is properly cited.

**Table of Contents**

Experimental Procedures ........ 1
Supporting Figures and Tables ........ 4
References ........ 23
Author Contributions ........ 23

## Experimental Procedures

Methods: Quantitative solubility of the xDEPPs in the electrolyte was also evaluated with UV-Vis measurements. The concentrations were obtained by using Beer's law (1) and the extinction coefficient that corresponds to the solvent (used electrolyte):

$$A = \varepsilon \cdot b \cdot c \qquad (1),$$

where A - absorbance, b - path length, c - concentration and ε - extinction coefficient.

A) Determination of molar extinction coefficients in the electrolyte.[1]

In the case of all used porphyrins, working stock solutions (in 1M $LiPF_6$ in PC:DMC:EC 1:3:1) were prepared with known concentrations. An appropriate amount of the xDEPPs were weighed with an Electrobalance in platinum crucibles with an uncertainty of ±5 mg and dissolved in 250 µL electrolyte. These samples were allowed to equilibrate for 12 h at 23 ± 2 °C. For each compound, we prepared a series of standard curve dilutions in order to determine the extinction coefficient out of the slope in a linear fit (2) (Table 1):

$$slope = \frac{\Delta c}{\Delta A} = \varepsilon b \qquad (2).$$

A linear correlation between absorbance and concentration for all compounds, demonstrates no aggregation occurs in solutions at the measured concentrations.

B) Solubility Determination.[1]

To prepare the saturated solution crystalline metalloporphyrin was introduced into a test tube containing 250 µL electrolyte. The addition was stopped when excess metalloporphyrin was visible at the bottom of the test tube. These samples were allowed to equilibrate for 12 h at 23 ± 2 °C. It was then passed through a 0.45-µ filter in order to remove any solid particles. Concentrations of saturated solutions were too high to measure absorbances directly and up to 10-fold dilutions were necessary. The concentrations were then obtained by using Beer's law (2) and the extinction coefficient that corresponds to the solvent (e.g. electrolyte).

Saturated solutions are giving us a trend for solubility in the electrolyte, which is fitting exactly our performance tests.

*Synthesis:* The synthesis of A2B2-porphyrins is straightforward and consists of three synthesis steps (Scheme 1). In step one, we synthesize the appropriate *meso*-dipyrromethane from pyrrole and an aldehyde with the intended substituent with the electron-withdrawing group. In step two, the ring-closing reaction takes place, flowing the Macdonald condensation. The condensation takes place between the *meso*-dipyrromethane and (trimethylsilyl)-propiolaldehyde. The metalation of the free-base porphyrin is unproblematic – conditions depend on the used metal.

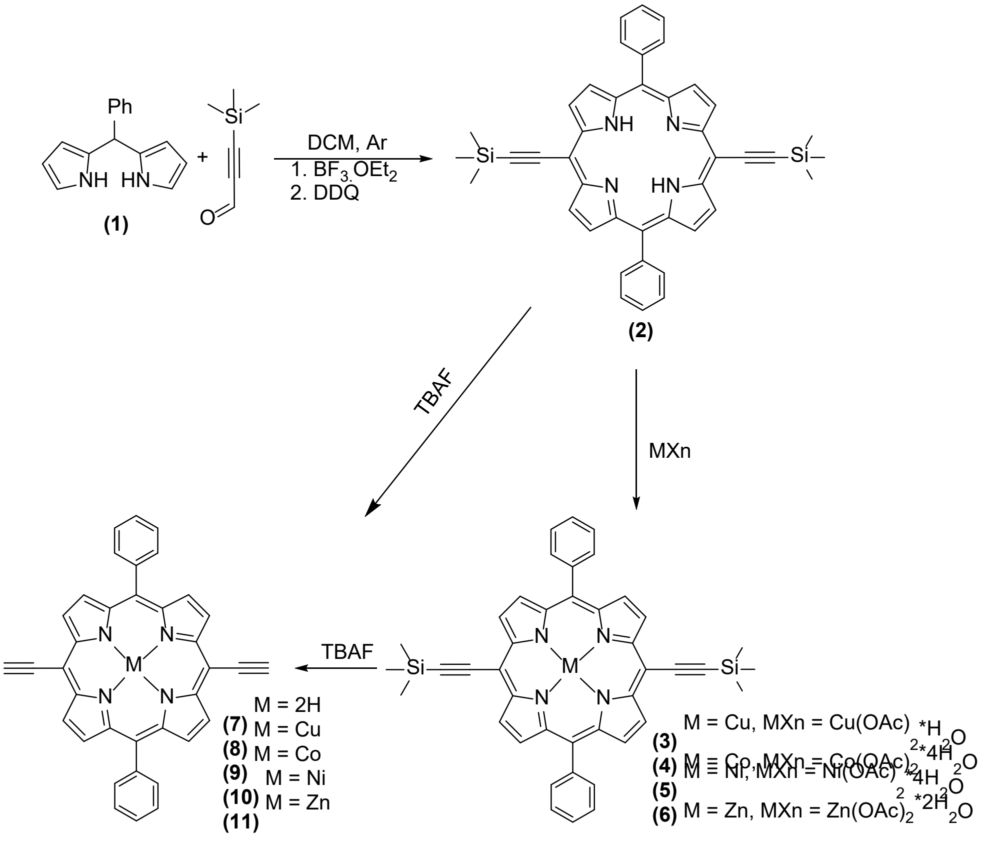


**Scheme 1**: Synthesis route.

**Synthesis of 5-phenylpyrromethane (1).**[2] A mixture of pyrrole (140 mL, 2 mol) and benzaldehyde (10.2 mL, 0.1 mol) was bubbled 15 min with Argon. The reaction mixture was cooled with an ice bath and trifluoroacetic acid (0.78 mL, 0.01 mol) was added dropwise. After that, the reaction mixture was extracted 3x with ethyl acetate. The combined organic phase was extracted with water and dried over sodium sulphate. After column chromatography ($SiO_2$, hexane:ethyl acetate, 2:1) , a yellow solid was obtained with 9% (2.0 g) yield. $^1$H-NMR (500 MHz, $CDCl_3$): 7.95 (2H, s, NH), 7.27-7.39 (5H, m, Ph), 6.73-6.74 (2H, m, CH pyrrole), 6.22 (2H, q, J = 2.9 Hz, CH pyrrole), 5.96-5.98 (2H, m, CH pyrrole) ppm, 5.52 (1H, s, CH).

**Synthesis of 5,15-bis(trimethylsilylethynyl)-10,20-diphenyl-21*H*, 23H-porphyrin (DEPP-TMS) (2).**[3] A mixture of (1) (1 g, 4.5 mmol) and 3-(trimethylsilyl)-2-propynal (0.69 mL, 4.7 mmol) in DCM

was cooled in an ice bath and bubbled for 15 min with Argon. After $BF_3{\cdot}OEt_2$ (0.08 mL, 0.68 mmol) was added dropwise, the reaction mixture was allowed to warm up to room temperature. DDQ (0.86 g, 3.8 mmol) was added and the mixture was stirred for 1 h. The reaction was quenched with TEA and filtrated. The solvent was removed under vacuum and after column chromatography ($SiO_2$, hexane: dichloromethane, 1:1), a blue solid was obtained with a yield of 45% (0.67 g). $^1$H-NMR (500 MHz, $CDCl_3$): 9.61 (4H, d, J = 4.5 Hz, CH pyrrole), 8.83 (4H, d, J = 4.45 Hz, CH pyrrole), 8.18 (4H, d, 7.45 Hz, Ph), 7.45-7.81 (6H, m, Ph), 0.60 (18H, s, TMS), -2.20 (2H, s, NH) ppm.

**[5,15-bis(trimethylsilylethynyl)-10,20-diphenylporphinato]cupper(II) (CuDEPP-TMS) (3).**[3] The free-base porphyrin (2) (0.52 g, 0.8 mmol) and $Cu(OAc)_2{\cdot}H_2O$ (0.52 g, 2.6 mmol) were dissolved in 70 mL DCM and 7 mL methanol and stirred overnight. The reaction mixture was filtered through celite. After a flash column chromatography ($SiO_2$, DCM), the product was obtained as a dark blue-purple solid with a yield of 64%. **$^1$H-NMR** (paramagnetic compound). **Elemental analysis** calcd for $C_{42}H_{36}CuN_4Si_2$: C 70.41 H 5.06 N 7.82 found: C 72.85 H 4.75 N 7.84

**[5,15-bis(trimethylsilylethynyl)-10,20-diphenylporphinato]zinc(II) (ZnDEPP-TMS) (6)[2].** The free-base porphyrin (0.23 g, 0.35 mmol) was dissolved in 125 mL dichloromethane and $Zn(OAc)_2{\cdot}2H_2O$ (0.27 g, 1.23 mmol) in 3 mL methanol was added. The mixture was stirred at room temperature overnight. The solvent was removed under reduced pressure; a dark green solid was obtained with a yield of 83% (0.19 g). **$^1$H-NMR (500 MHz, $CDCl_3$)**: 0.60 (18H, s, TMS), 7.77-7.79 (6H, m, Ph), 8.18 (4H, d, J = 6.25 Hz, Ph), 8.92 (4H, d, J = 4.55 Hz, β-H), 9.70 (4H, d, J = 4.55 Hz, β-H) ppm. **Elemental analysis** calcd for $C_{42}H_{36}ZnN_4Si_2$: C 70.23 H 5.05 N 7.80 found: C 69.75 H 4.90 N 7.53

**General procedure for deprotection of TMS-group.**[4] The porphyrin (0.05 mmol) was dissolved in 20 mL dry THF and 1 mL of 1M solution of TBAF in THF was added. The mixture was stirred overnight under an argon atmosphere. The reaction was quenched by adding 50 mL of water. THF was removed under reduced pressure. The precipitate was filtrated and dried overnight at 100 °C and $2.0{\cdot}10^{-2}$ mbar.

**5,15-bis(ethynyl)-10,20-diphenylporphin (DEPP) (7).**[5]

**MALDI-ToF-MS**: calculated for $C_{36}H_{22}N_4$ $[M]^+$. m/z: 511.3; found 511.2 (100%) $[M]^+$.

**[5,15-bis(ethynyl)-10,20-diphenylporphinato]copper(II) (CuDEPP) (8).**[3]

**Elemental analysis** calcd for $C_{36}H_{20}CuN_4$: C 75.58 H 3.52 N 9.79 found: C 74.73 H 3.75 N 9.18.

**[5,15-bis(ethynyl)-10,20-diphenylporphinato]zinc(II) (ZnDEPP) (11).**[6]

**MALDI-ToF-MS**: calculated for $C_{36}H_{20}N_4Zn$ $[M]^+$: m/z: 573.1; found 573.0 (100%) $[M]^+$. **Elemental analysis** calcd for $C_{36}H_{20}ZnN_4$: C 75.34 H 3.51 N 9.76 found: C 74.16 H 3.69 N 9.28.

## Supporting Figures and Tables

Matrix-assisted laser desorption and ionization time-of-flight mass spectrometry (MALDI ToF MS) is frequently applied to analyze macrocycles and their metal complexes. All MALDI mass spectra were obtained by solvent-based sample preparation methods. About 0.1 mg of the analyte was dissolved or suspended in 2 ml of MeOH. A small amount (0.1-2.5 μL) of the solution was put on the stainless steel substrate and dried in air.

The MALDI-TOF mass spectra of newly synthesized **4, 5, 9, 10** showed appropriate signals for the molecular ions and proved their desired nature. The molecular ion was the most abundant high mass ion with a distinct isotopic distribution in all cases. The relative abundances of the isotopic ions are in good agreement with the simulated spectra, as reported in **Figure S3-S6**, where every spectral result and calculated values are summarised. In all cases except NiDEPP (**10**), mass spectra were detected in the positive ion mode. In addition, acidic compounds (such as alkynes) can also be detected as single negatively charged ions in the negative ion MALDI ToF mass spectra.

It is common for small ions like $Ni^{2+}$ that insertion into porphyrins suffers from the fact that $Ni^{2+}$ is too small to perfectly fit into the square planar cavity formed by the four pyrrole nitrogen atoms (ionic radii $Mg^{2+} \cong Co^{2+} \cong Cu^{2+} \cong Zn^{2+} > Ni^{2+}$) (**Figure S1**) $Ni^{II}$-porphyrins show a rich conformational behavior: a $(d_{z2})^2$ electronic configuration and small ionic radius (0.69 Å) of $Ni^{II}$ favor relatively short equilibrium Ni-N bond distances. This results in nonplanar ruffled Ni-porphyrin conformations,[7] in which individual pyrrole rings are twisted about the Ni-N axes and significant alternating displacements of the $C_m$ sites above and below the mean molecular plane take place.[8–10]

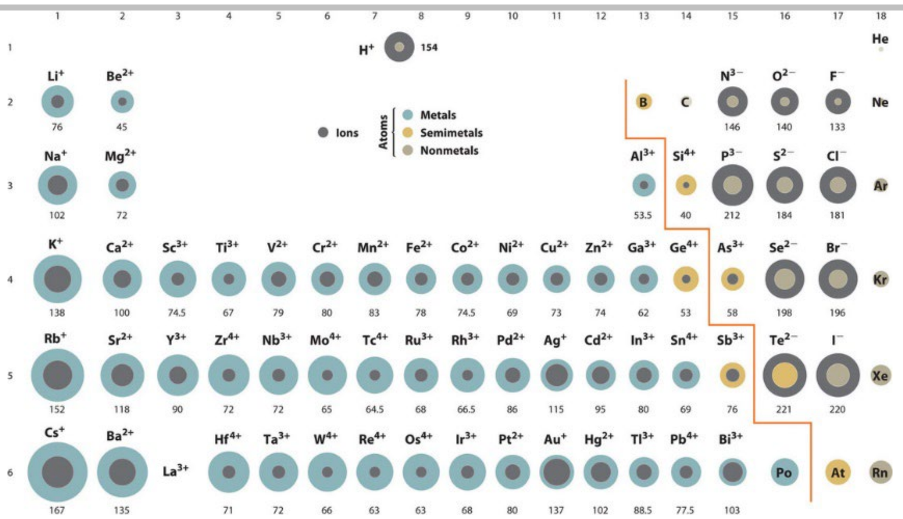


**Figure S1.** Calculated ionic radii (in picometers) of the most common ionic states of the *s*-, *p*-, and *d*-block elements. Gray circles indicate the sizes of the ions shown; colored circles indicate the sizes of the neutral atoms. The calculated values are based on quantum mechanical wave functions.[11] Source: http://chem.libretexts.org

The degree of nonplanarity of metalloporphyrins depends as well on such factors as the substituent size, shape, and orientation, pyrrole nitrogen(s) substitution by other elements, etc. Considering all those facts, the different results of MALDI ToF MS for NiDEPP (**10**) can be explained as follows: the absence of bulky TMS-groups facilitates electron capture by the analyte molecule via two pathways: (a) by central metal ion $Ni^{2+}$, resulting in its reduction to $[Ni^{0}M^{-}]$; and (b) by the formation of vinyl anion-radical,[12] its transformation in gas phase due to addition of alkali metal ions -commonly observed impurities for samples prepared by the solution- based methods, and protons followed by elimination of $–CH_3$ fragment (Fig. S2, S4). This cascade of transformations is evidenced by the MALDI-TOF mass spectra showing an intense signal for the molecular ion $[(M+3H^{+}-CH_3)^{3-}+ Li^{+}+H^{+}]^{-}$ at $m/z$ = 561,1052 with 561,0822 $[M^{-}]$ calculated for $C_{35}H_{21}N_4Li$.

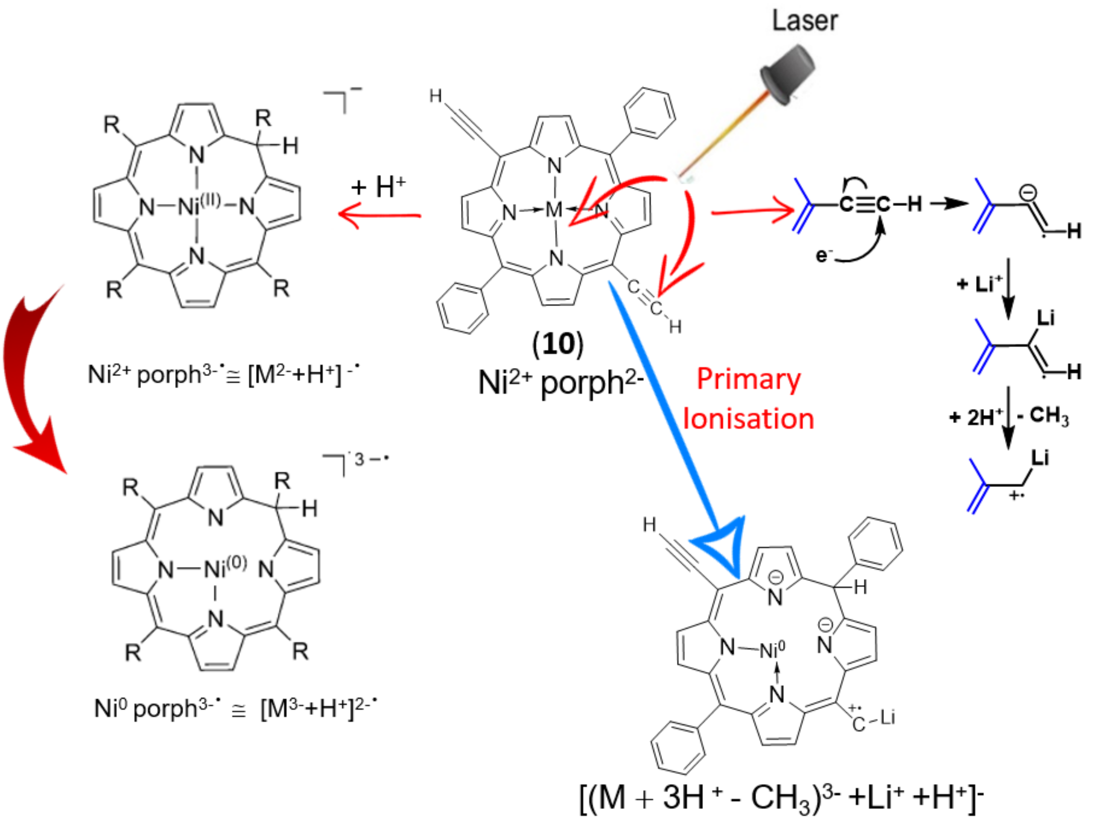


**Figure S2**. Proposed mechanism of molecular ion $[(M+3H^{+}-CH_3)^{3-}+ Li^{+}+H^{+}]^{-}$ formation upon electron capture by NiDEPP (**10**) in the negative ion mode via primary ionization.

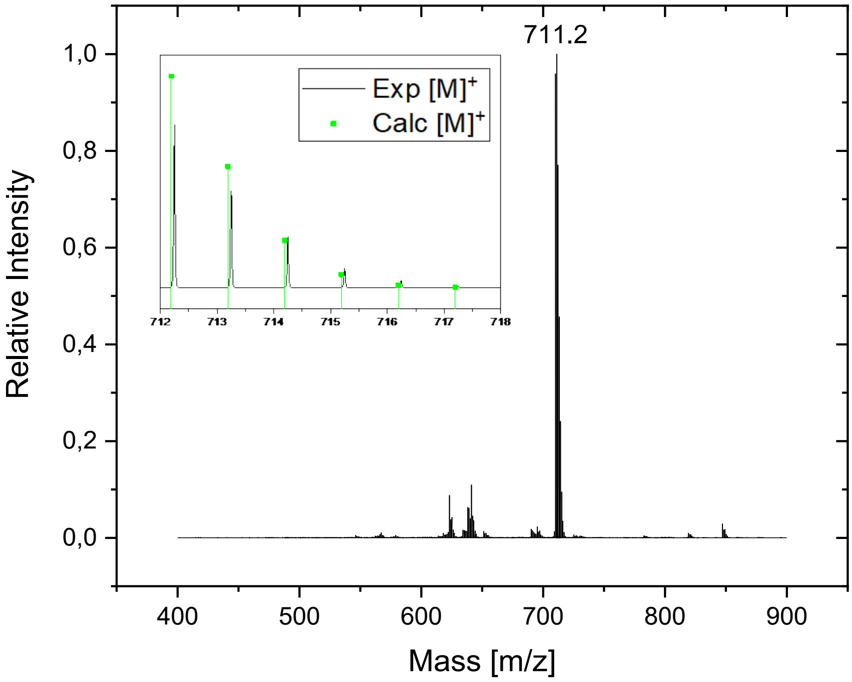

**Figure S3**: MALDI-ToF of NiDEPP-TMS **(5)**.

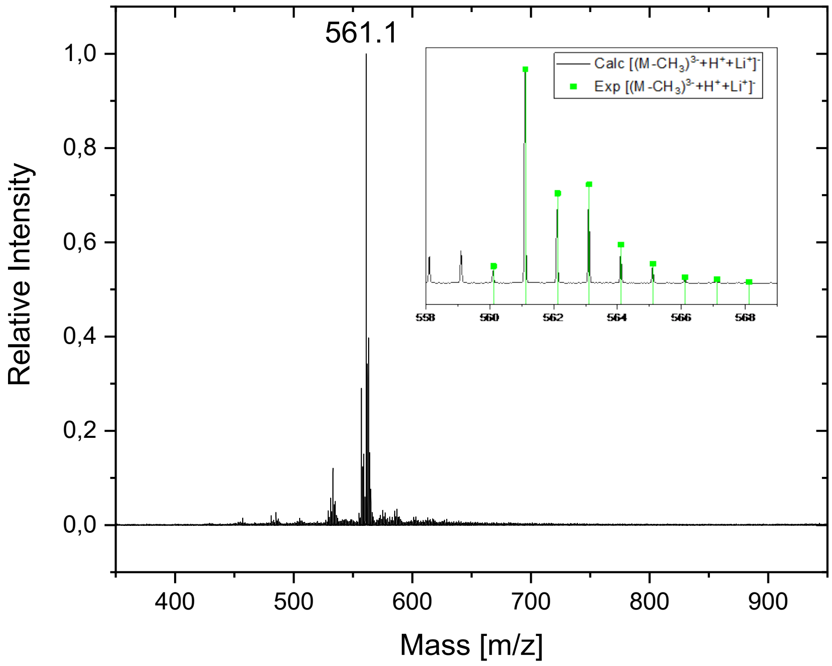


**Figure S4**: MALDI-ToF of NiDEPP **(10)**.

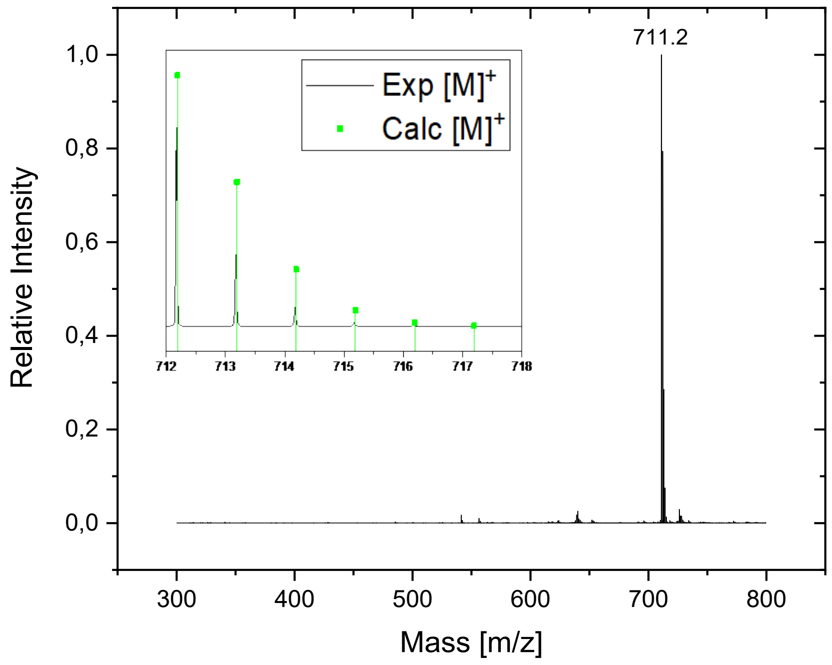


**Figure S5**: MALDI-ToF of CoDEPP-TMS **(4)**.

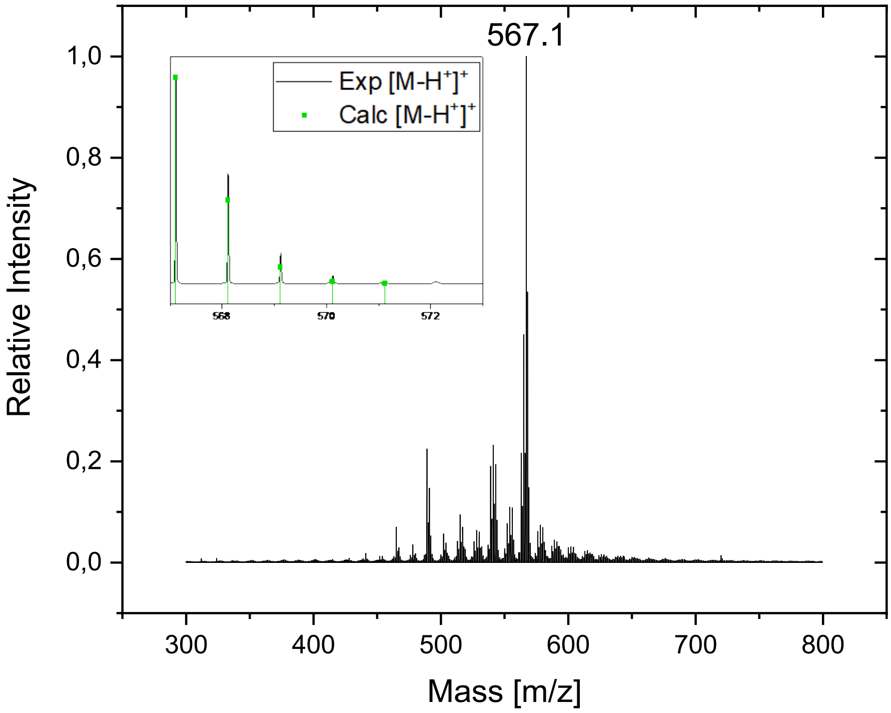


**Figure S6**: MALDI-ToF of CoDEPP **(9)**.

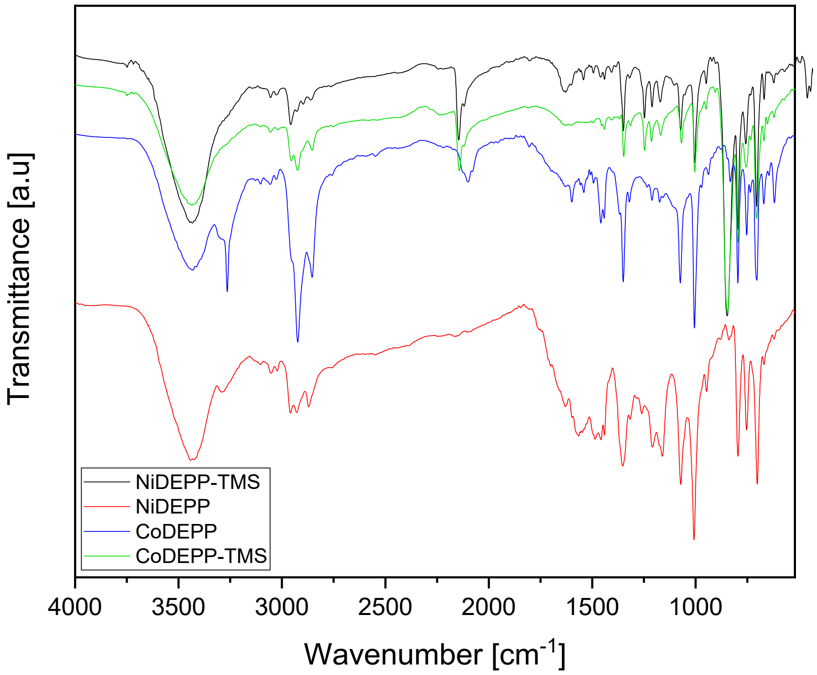


**Figure S7**: IR spectra of 4-5 and 9-10.

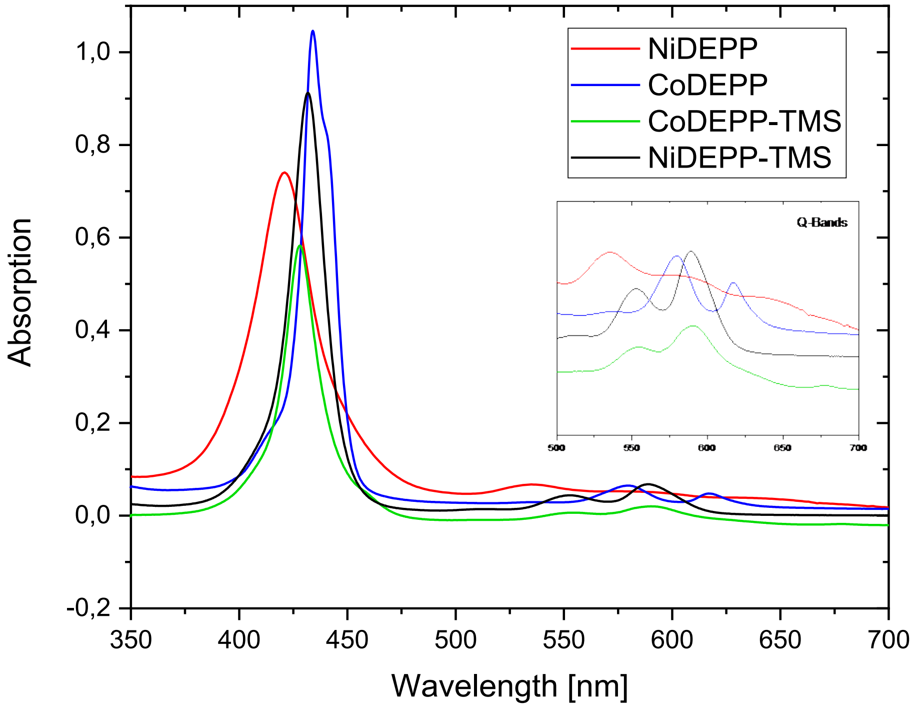


**Figure S8**: UV-Vis spectra of 4-5 and 9-10.

**Table S1**. $\varepsilon$ and concentration of saturated solutions of xDEPPs in electrolyte after 1 day confirming the limited solubility of CuDEPP and very high solubility of NiDEPP.

| | ε [L/(mol·cm)] | Concentration [mol/L] | Concentration [g/L] |
|---|---|---|---|
| **DEPP** | 3787.27 | 0.11259 | 57.49 |
| **CoDEPP** | 5107.10 | 0.01055 | 5.99 |
| **NiDEPP** | 1393.33 | 0.02638 | 14.96 |
| **CuDEPP** | 35.20 | 0.00345 | 1.97 |
| **ZnDEPP** | 10595.64 | 0.00492 | 2.82 |

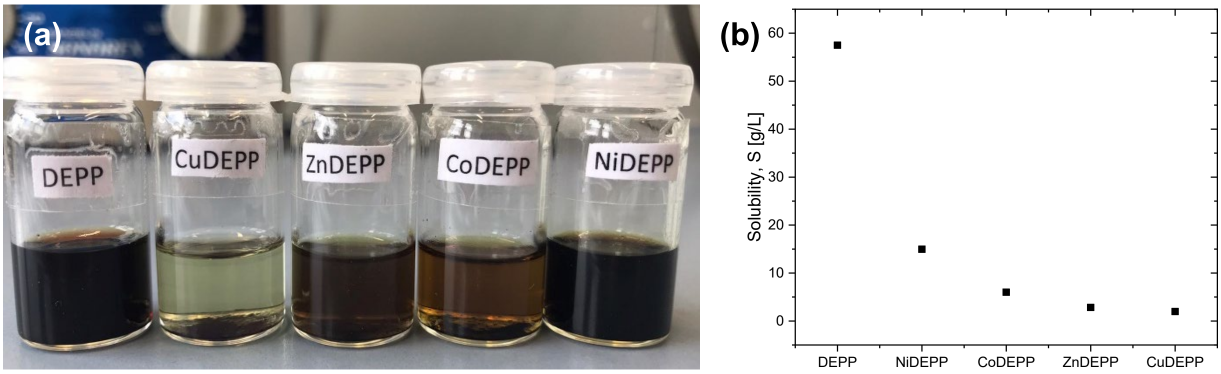


**Figure S9.** Visual comparison of the solubility of xDEPPs in dichloromethane after 1day confirming the limited solubility of CuDEPP and very high solubility of NiDEPP (a). Saturated solutions trend for the solubility of xDEPPs in the electrolyte (b).

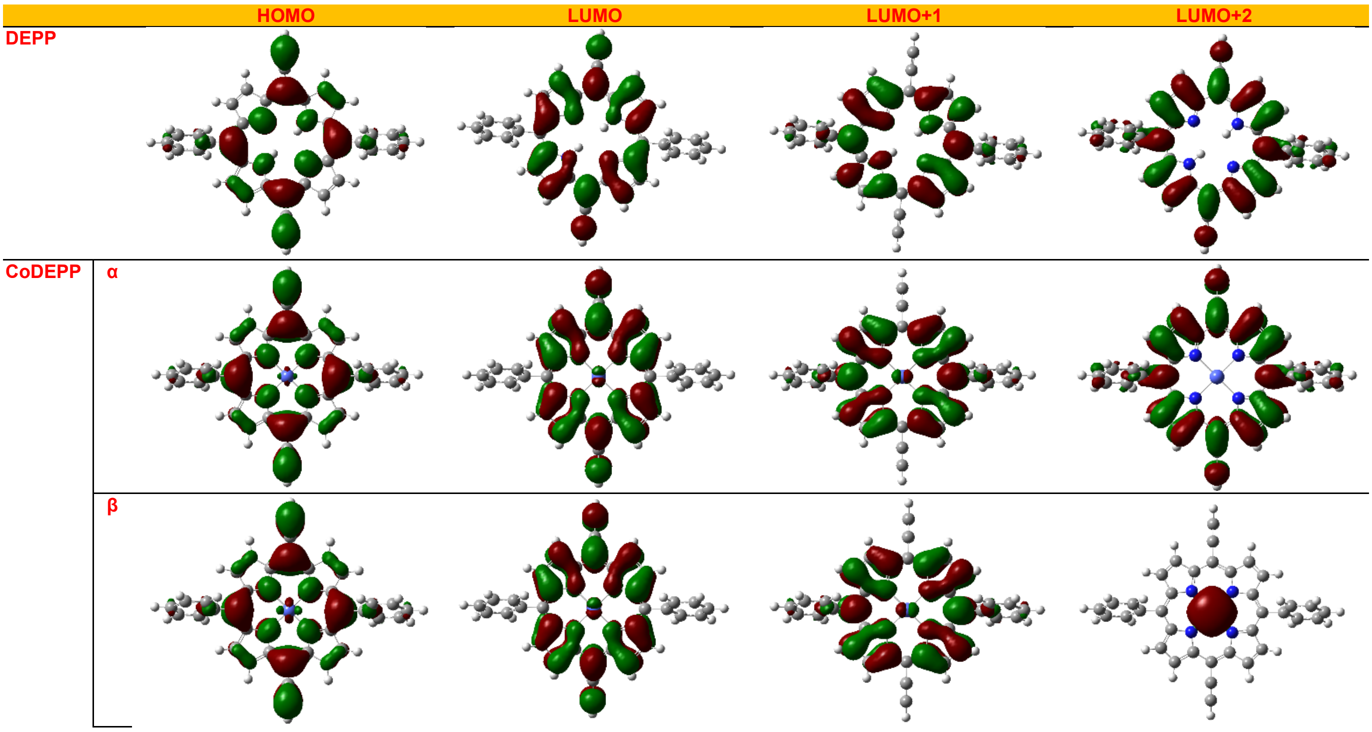
HOMO
LUMO
LUMO+1
LUMO+2
DEPP
CoDEPP
α
β

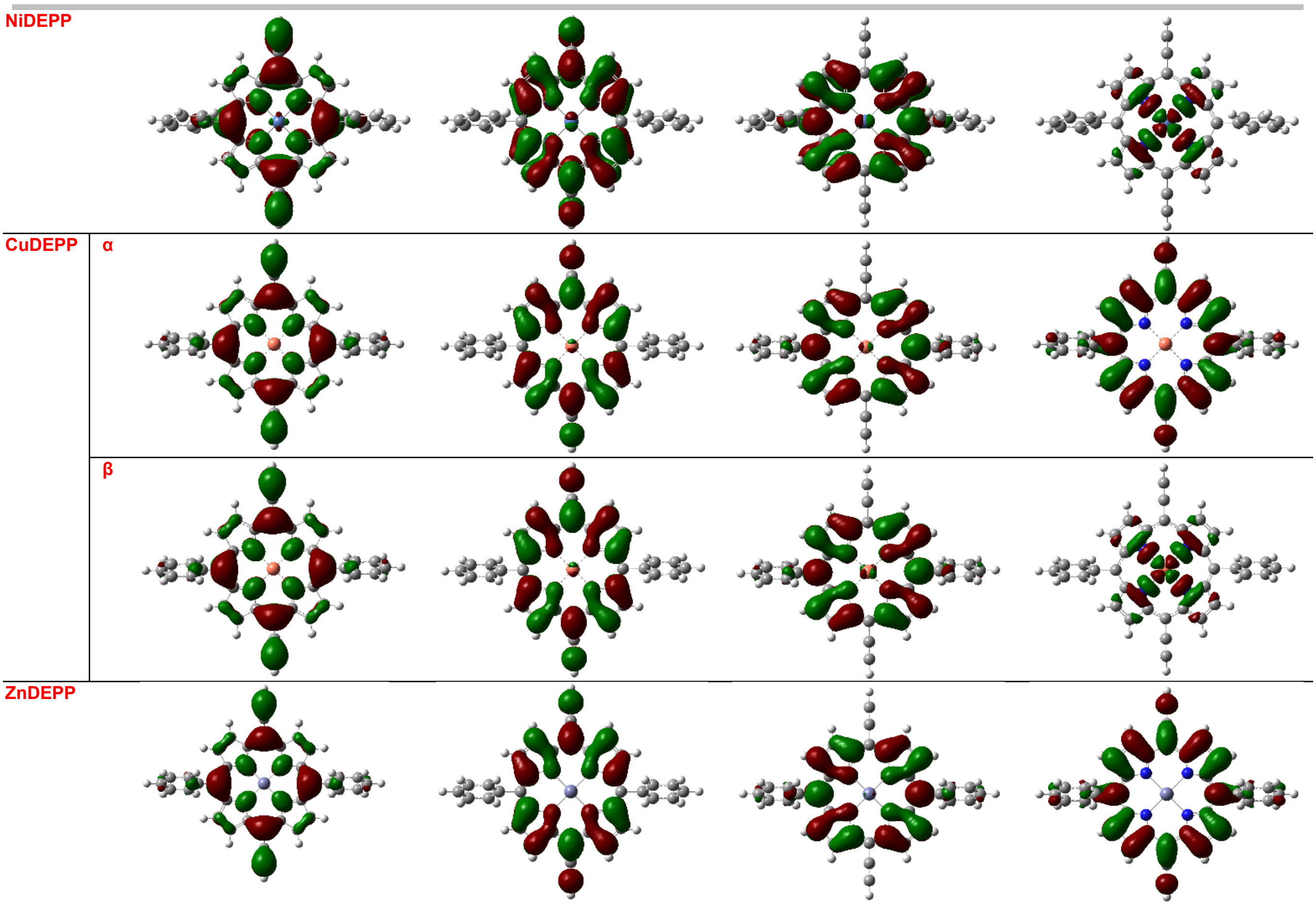


**Figure S10.** Frontier orbitals (highest occupied molecular orbitals and lowest unoccupied molecular orbitals) of xDEPPs. LUMO+1 and LUMO+2 denote the molecular orbitals above the LUMO.

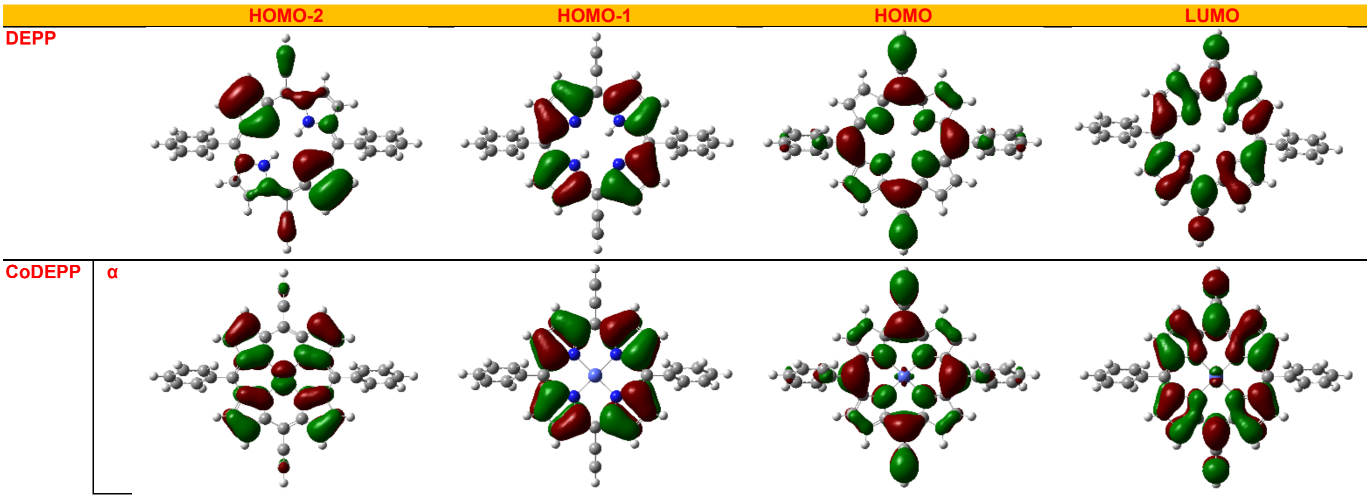

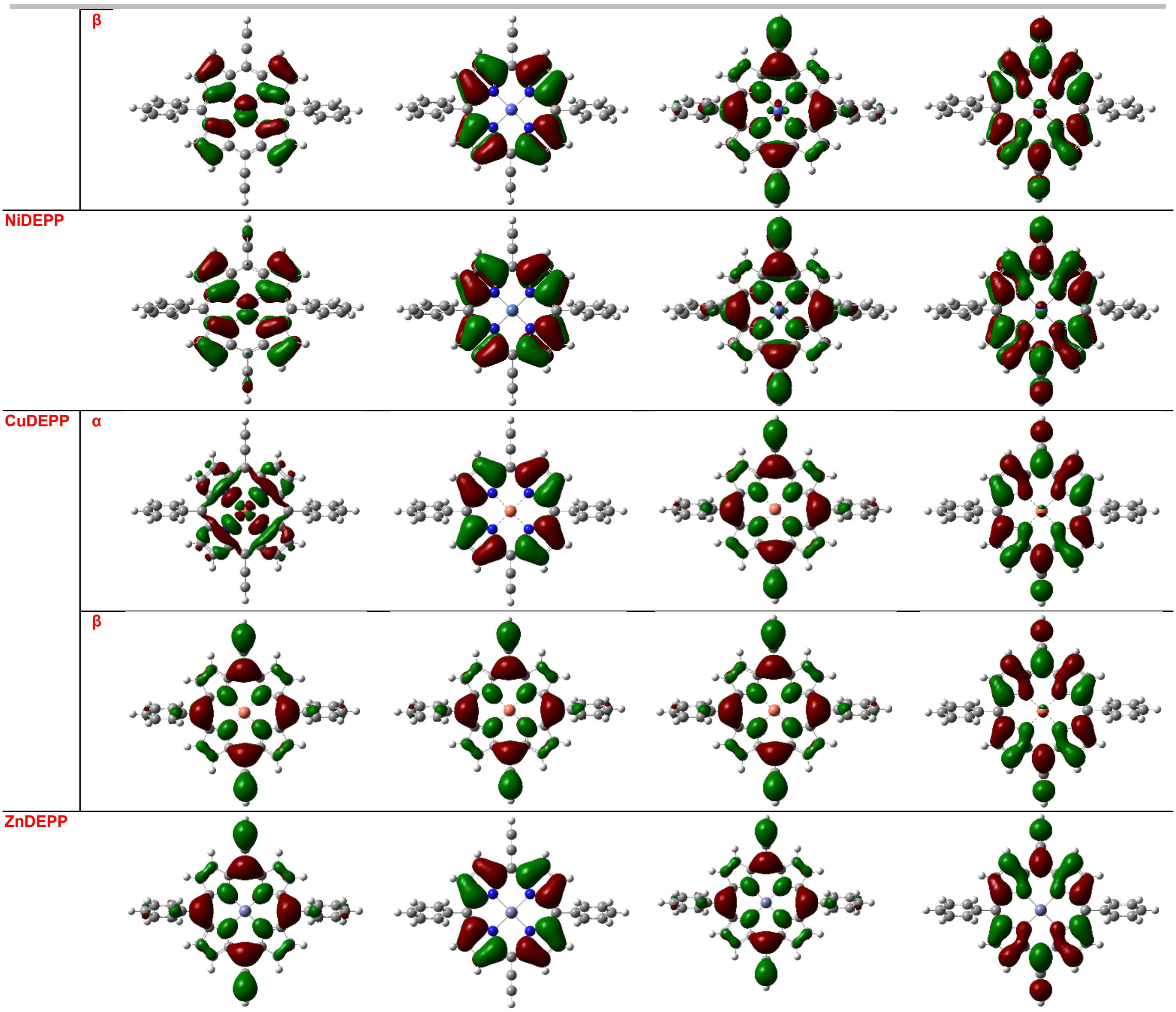


**Figure S11.** Frontier orbitals (highest occupied molecular orbitals and lowest unoccupied molecular orbitals) of xDEPPs. HOMO-2 and HOMO-1 denote the molecular orbitals below the HOMO.

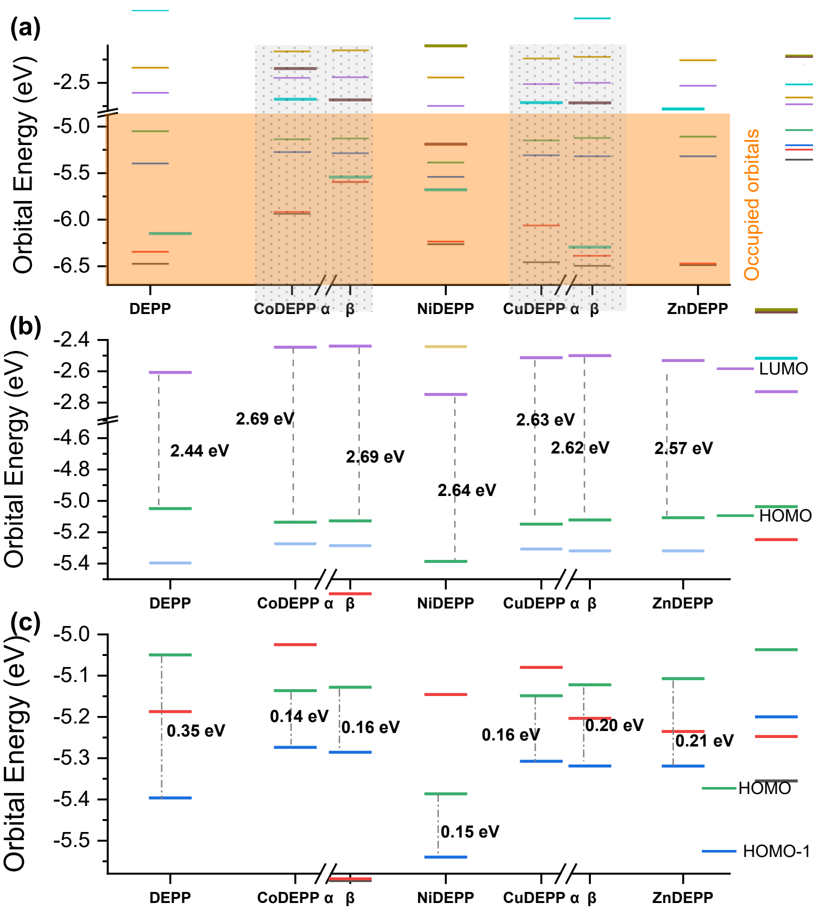


**Figure S12.** Schematic drawings of (a) energies of frontier molecular orbitals of xDEPPs, and (b) HOMO-LUMO and (c) HOMO-HOMO-1 energy gap of xDEPPs.

**Table S2**. Calculated and experimental bond lengths (Å) in xDEPPs.

| | | CoDEPP | NiDEPP | CuDEPP | ZnDEPP |
|---|---|---|---|---|---|
| **x–N** | Calc. | 1.964, 1.961 | 1.934, 1.937 | 2.044, 2.037 | 2.043 |
| | Exp. | 1.959, 1.957, 1.962, 1.964 | - | 1.996, 2.002 | - |
| **N–C** | Calc. | 1.393, 1.388, 1.397, 1.389 | 1.371, 1.379, 1.380 | 1.381, 1.374 | 1.379, 1.371 |
| | Exp. | 1.385, 1.377, 1.384, 1.376 | - | 1.363, 1.368, 1.381, 1364 | - |

**Table S3**. Solubility of Co and Cu complexes upon cycling.

| Solubility of | Pristine materials in the electrolyte (g/L) | Cells kept at OCV for 10 h (g/L) | Cells after 1 cycle (g/L) | Cells after 100 cycles (g/L) |
|---|---|---|---|---|
| CoDEPP | 5.99 | 2.1 | n.d* | 2.4 |
| CuDEPP | 1.97 | n.d* | n.d* | n.d* |

*n.d refers to the very low solubility of the electrolyte (under the detection limit)

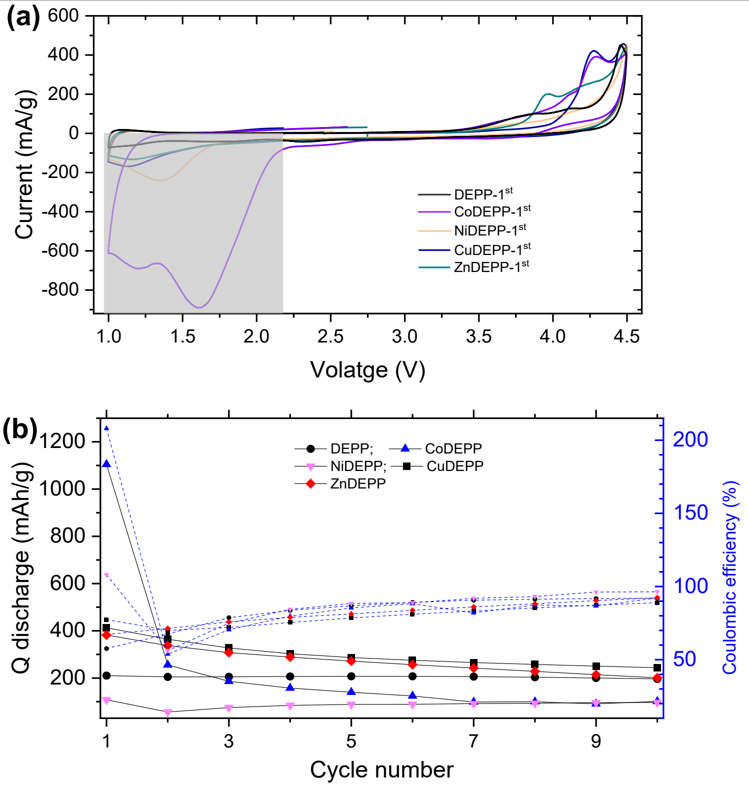


**Figure S13.** Comparison of the electrochemical properties of the xDEPPs electrodes: (a) the first CV curves obtained at 0.1 mV $s^{-1}$ and (b) discharge capacity and CE of xDEPPs in the initial 10 cycles based on the CV measurement in the potential range of 1-4.5 V (Figure 2b-e).

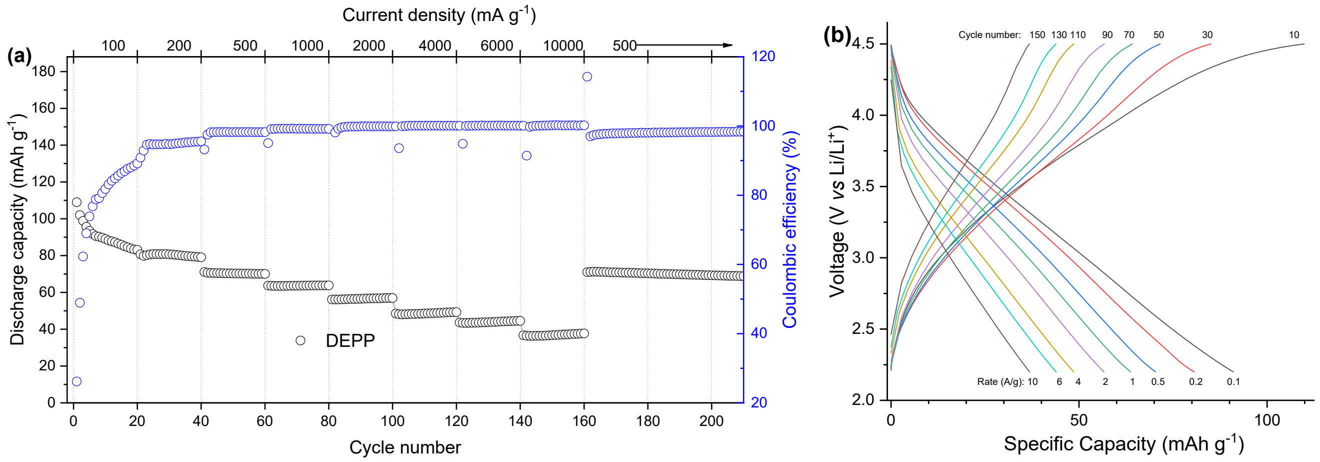


**Figure S14.** The rate capability of DEPP electrode with an increase in the charge-discharge rate from 100 mA $g^{-1}$ to 10 A $g^{-1}$ and then a decrease to 500 mA $g^{-1}$(a) and selected voltage profile (b).

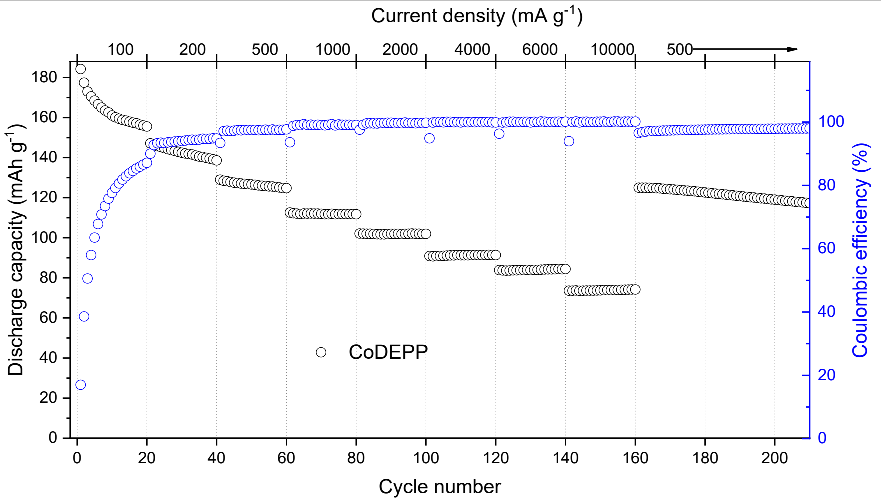


**Figure S15.** The rate capability of CoDEPP electrode with an increase in the charge-discharge rate from 100 mA $g^{-1}$ to 10 A $g^{-1}$ and then a decrease to 500 mA $g^{-1}$.

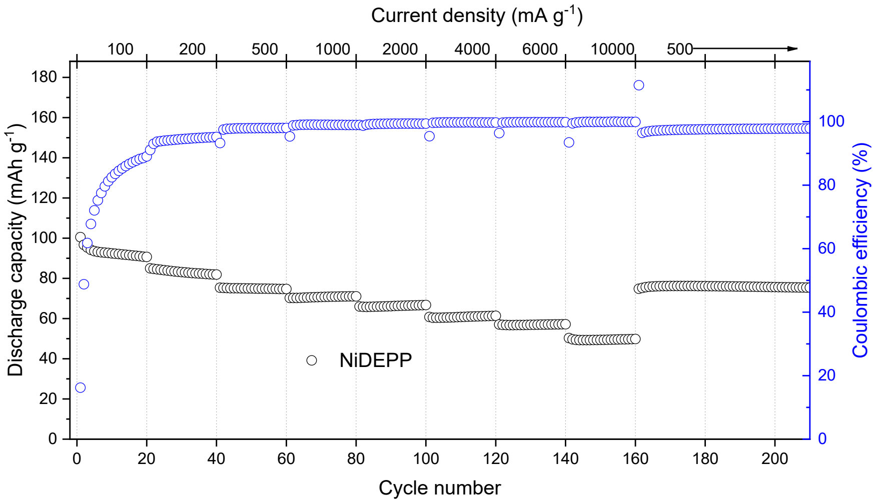


**Figure S16.** The rate capability of NiDEPP electrode with an increase in the charge-discharge rate from 100 mA $g^{-1}$ to 10 A $g^{-1}$ and then a decrease to 500 mA $g^{-1}$.

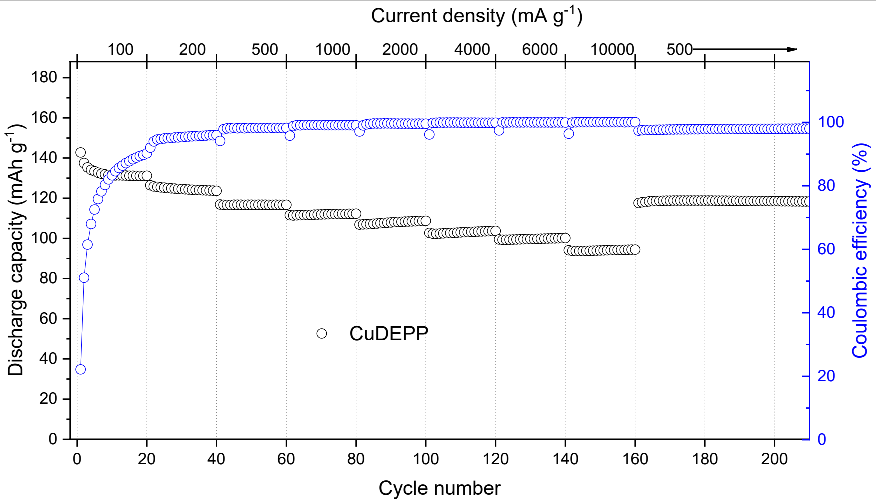


**Figure S17.** The rate capability of CuDEPP electrode with an increase in the charge-discharge rate from 100 mA $g^{-1}$ to 10 A $g^{-1}$ and then a decrease to 500 mA $g^{-1}$.

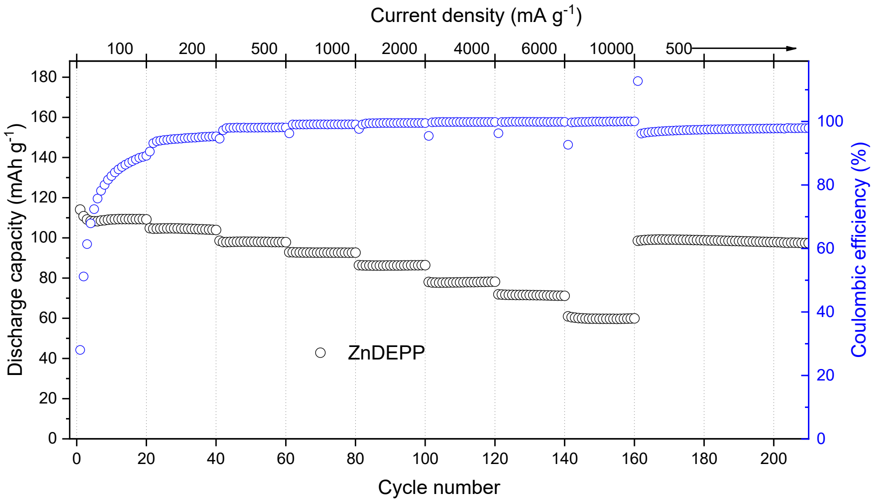


**Figure S18.** The rate capability of ZnDEPP electrode with an increase in the charge-discharge rate from 100 mA $g^{-1}$ to 10 A $g^{-1}$ and then a decrease to 500 mA $g^{-1}$.

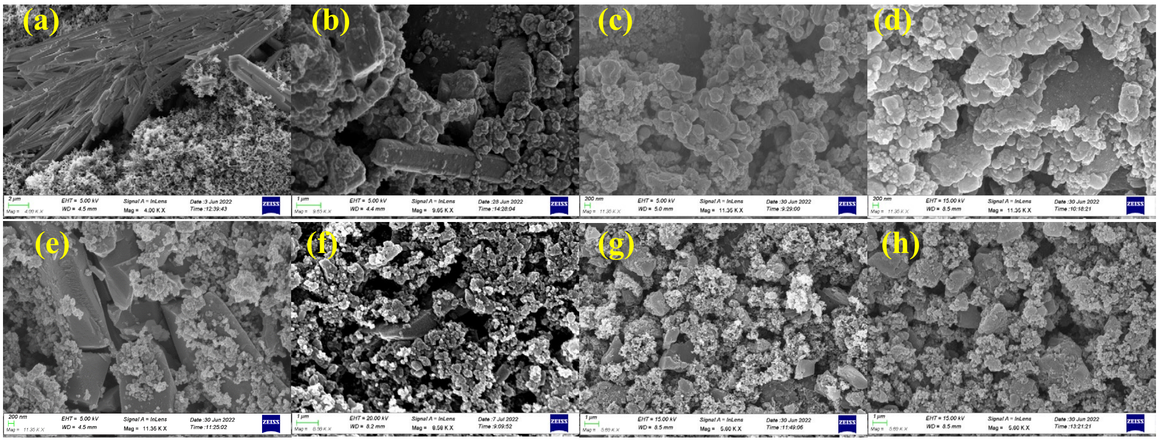


**Figure S19.** Morphology of the as prepared metal DEPP electrodes (a, c, e, g), and after 100 cycles (b, d, f, h) for CuDEPP (a, b), CoDEPP (c, d), ZnDEPP (e, f), and NiDEPP (g, h).

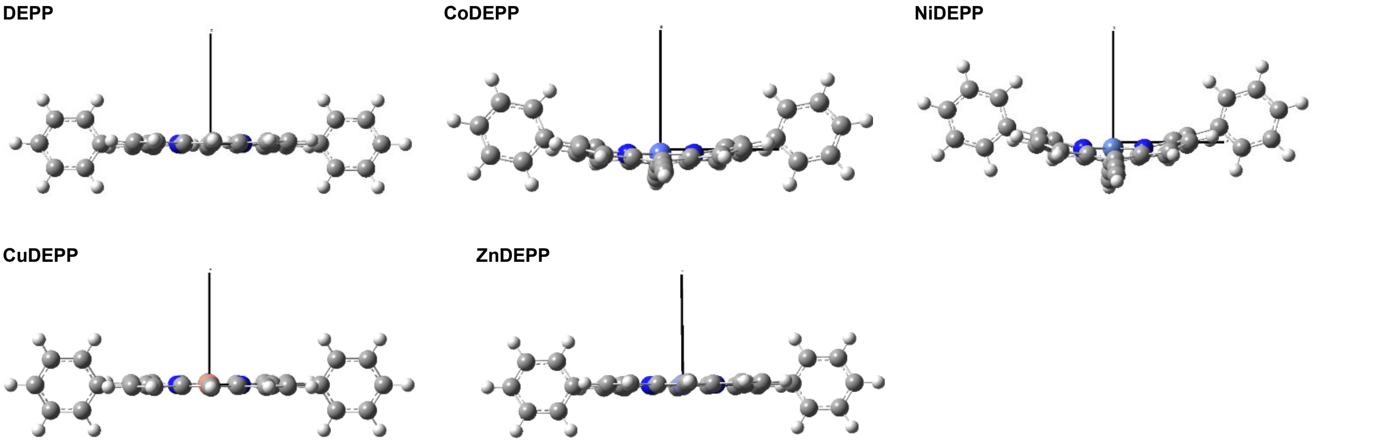


**Figure S20.** The DFT-optimized structures of xDEPPs showing saddled distortion for NiDEPP and CoDEPP.

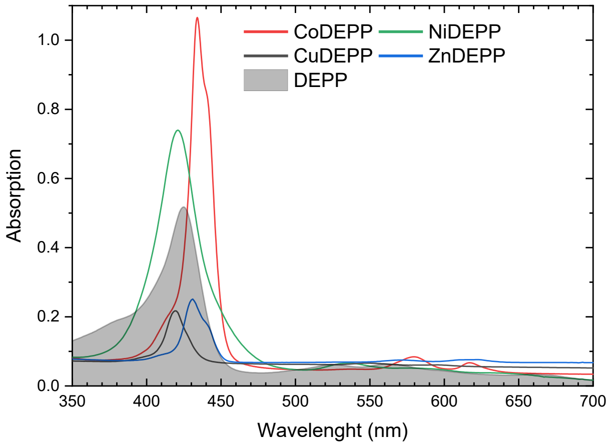


**Figure S21.** UV/Vis absorption spectra of xDEPPs.

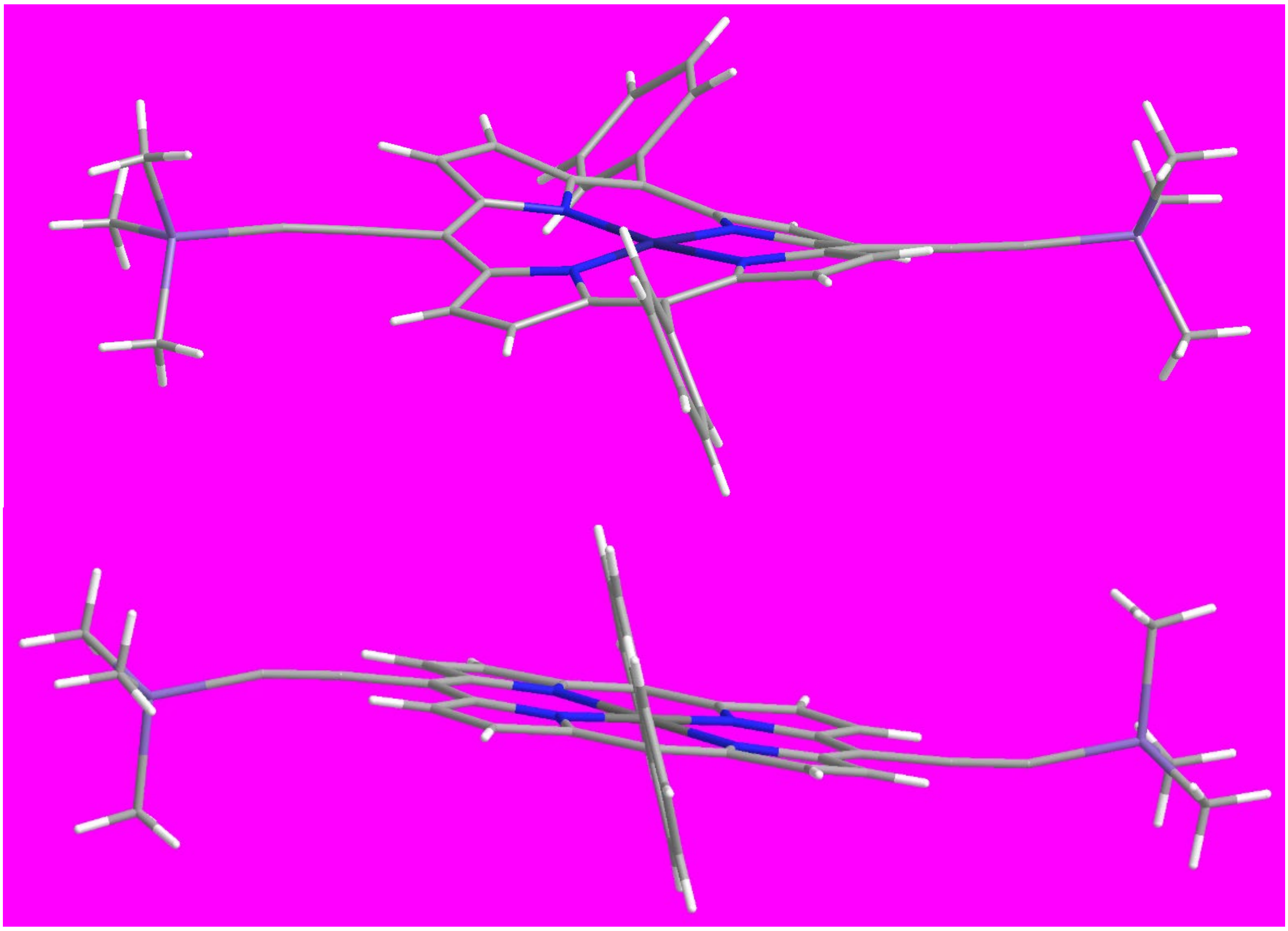

**Figure S22.** Comparative crystal structures of CoDEPP-TMS (top) and CuDEPP-TMS [3] (bottom).

**Table S4**: Crystal data and structure refinement.

| | **CoDEPP-TMS (4)** | **CuDEPP-TMS (3)**[3] |
|---|---|---|
| **Empirical formula** | $C_{42}H_{36}N_4Si_2Co$ | $C_{42}H_{36}N_4Si_2Cu$ |
| **Formula weight/g*mol$^{-1}$** | 711.86 | 716.47 |
| **Temperature/K** | 150 | 180.15 |
| **Crystal system** | Triclinic | Triclinic |
| **Space group** | $P\bar{1}$ | $P\bar{1}$ |
| ***a***/Å | 9.3678(4) | 6.0685(5) |
| ***b***/ Å | 14.0547(7) | 11.9579(10) |
| ***c***/ Å | 15.0024(7) | 12.7655(10) |
| **α/°** | 66.824(5) | 76.138(6) |
| **β/°** | 82.406(4) | 81.136(7) |
| **γ/°** | 79.768(4) | 84.850(7) |
| **Volume/ Å$^3$** | 1782.73(16) | 887.28(13) |
| ***Z*** | 2 | 1 |
| **$\rho_{calc}$g/cm$^3$** | 1.326 | 1.341 |
| **$\mu$/mm$^{-1}$** | 0.585 | 0.719 |
| ***F*(000)** | 742 | 373.0 |
| **Cystal size/ mm$^3$** | 0.228x0.205x0.156 | 0.36x0.06x0.02 |
| **Radiation** | Mo Kα (λ = 71073) | Mo Kα (λ = 71073) |
| **2θ range for data collection/°** | 4.4 to 54.2 | 4.24 to 51.3 |
| **Goodness-of-fit on $F^2$** | 1.065 | 0.998 |
| **Final R indexes [$I \geq 2\sigma$ (I)]** | $R_1$= 0.0423 w$R_2$ = 0.1144 | $R_1$= 0.0543 w$R_2$ = 0.1121 |
| **CCDC number** | 2143452 | 1506859 |

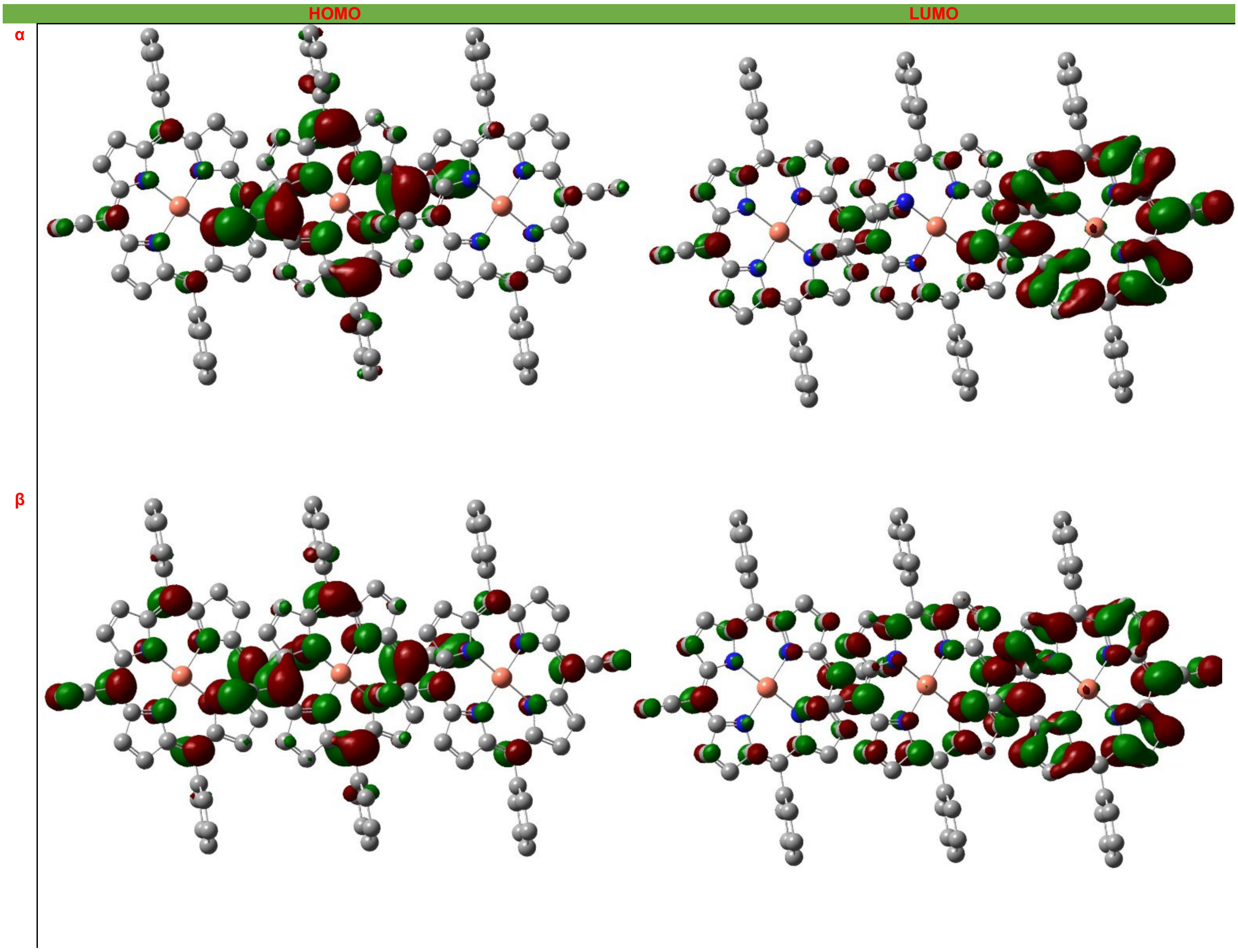


**Figure S23.** Frontier orbitals of $(CuDEPP)_3$.

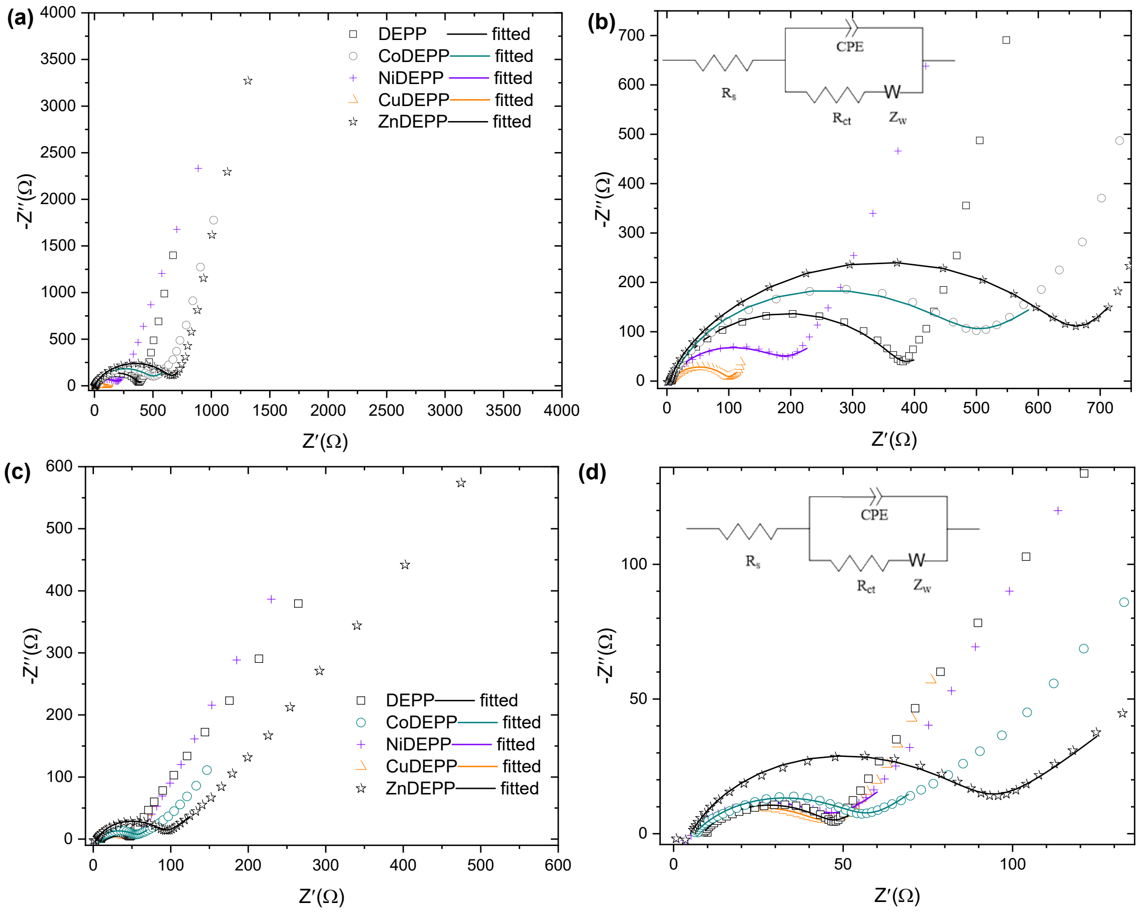


**Figure S24.** EIS of the xDEPP electrodes before (a) and after cycling at 100 mAh $g^{-1}$ (c) with enlarged part of the high frequency region (b, d) and the equivalent circuit model (inset).

**Table S5.** EIS analyses results of the cell based on the xDEPP cathodes (fresh cell and after 10 cycles).

| | $R_s$ (Ω) | | $R_{ct}$ (Ω) | | σ ($Ω.s^{-1/2}$) | |
|---|---|---|---|---|---|---|
| | Fresh cell | Cycled cell | Fresh cell | Cycled cell | Fresh cell | Cycled cell |
| DEPP | 7.0 | 8.5 | 361.9 | 38.5 | 235.9 | 58.3 |
| CoDEPP | 6.3 | 6.1 | 452.4 | 51.3 | 626.9 | 65.5 |
| NiDEPP | 4.3 | 5.1 | 167.4 | 42.0 | 501.2 | 85.4 |
| CuDEPP | 6.2 | 5.8 | 90.6 | 38.6 | 24.9 | 21.4 |
| ZnDEPP | 5.4 | 5.2 | 608.1 | 86.3 | 694.0 | 201.8 |

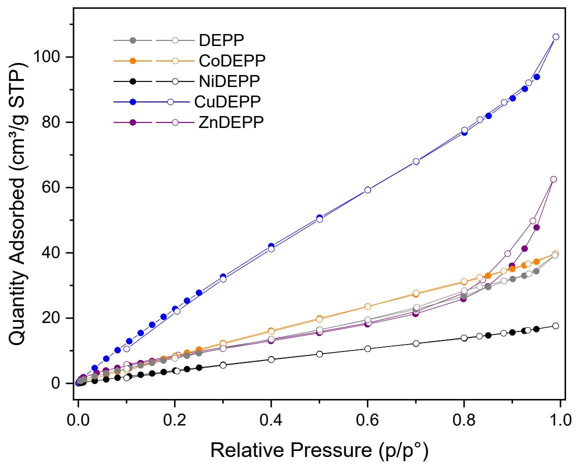


**Figure S25.** Nitrogen adsorption-desorption isotherms of the xDEPP active materials.

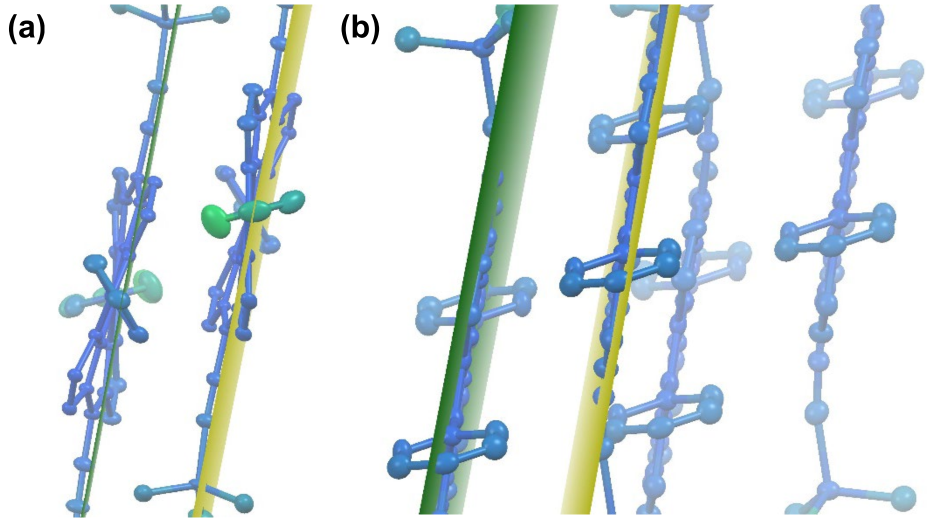


**Figure S26.** Molecular level stacking of CoDEPP (a) and CuDEPP (b).

**Table S6.** Thermal characterizations of xDEPPs

| | $T_{(5)}$[a] | $T_{(10)}$[b] | Wt%$_{@800\ °C}$[c] |
|---|---|---|---|
| **DEPP** | 268 | 320 | 56 |
| **CoDEPP** | 398 | 706 | 85 |
| **NiDEPP** | 244 | 277 | 64 |
| **CuDEPP** | 425 | 502 | 85 |
| **ZnDEPP** | 408 | 490 | 78 |

[a]Temperature of 5% weight loss, [b]temperature of 10% weight loss, and [c]the percentage of solid residue after heating from room temperature to 800 °C under argon.

## References


[1] D. H. Freeman, I. D. Swahn, P. Hambright, *Energy & Fuels* **1990**, *4*, 699–704.
[2] K. Zlatić, M. Cindrić, I. Antol, L. Uzelac, B. Mihaljević, M. Kralj, N. Basarić, *Org. Biomol. Chem.* **2021**, *19*, 4891–4903.
[3] P. Gao, Z. Chen, Z. Zhao-Karger, J. E. Mueller, C. Jung, S. Klyatskaya, T. Diemant, O. Fuhr, T. Jacob, R. J. Behm, M. Ruben, M. Fichtner, *Angew. Chemie Int. Ed.* **2017**, *56*, 10341–10346.
[4] I. Hijazi, T. Bourgeteau, R. Cornut, A. Morozan, A. Filoramo, J. Leroy, V. Derycke, B. Jousselme, S. Campidelli, *J. Am. Chem. Soc.* **2014**, *136*, 6348–6354.
[5] A. Ryan, A. Gehrold, R. Perusitti, M. Pintea, M. Fazekas, O. B. Locos, F. Blaikie, M. O. Senge, *European J. Org. Chem.* **2011**, *2011*, 5817–5844.
[6] P. K. Goldberg, T. J. Pundsack, K. E. Splan, *J. Phys. Chem. A* **2011**, *115*, 10452–10460.
[7] J. A. Shelnutt, *J. Porphyr. Phthalocyanines* **2000**, *04*, 386–389.
[8] M. K. Peters, R. Herges, *Inorg. Chem.* **2018**, *57*, 3177–3182.
[9] E. B. Fleischer, *Acc. Chem. Res.* **1970**, *3*, 105–112.
[10] R. G. Alden, B. A. Crawford, R. Doolen, M. R. Ondrias, J. A. Shelnutt, *J. Am. Chem. Soc.* **1989**, *111*, 2070–2072.
[11] R. D. Shannon, *Acta Crystallogr. Sect. A* **1976**, *32*, 751–767.
[12] P. R. Jenkins, M. C. R. Symons, S. E. Booth, C. J. Swain, *Tetrahedron Lett.* **1992**, *33*, 3543–3546.


## Author Contributions

*S.S.* synthesized the active electrode material (xDEPP), performed the material characterization, and contributed to the writing of the draft. *E.A-L.* supervised the electrochemical part, performed the electrochemical analysis, DFT calculations, and contributed significantly to the writing of the original draft. *J.C.* prepared and optimized the electrode compositions and collected the experimental cycling data. *T.D.* performed XPS analysis. *S.K.* was involved in the xDEPP characterization. *F.P.* was involved in DFT calculations. *A.M.* performed a single crystal analysis. *M.F.* and *M.R.* conceived the idea, supervised the work, and were involved in funding acquisition. All authors contributed to revising the paper.
